# WDR82 code coordinates transcription and pre-mRNA 3′-end processing through regulated phosphorylation

**DOI:** 10.1038/s41467-026-77611-z

**Published:** 2026-09-15

**Authors:** Alex Au, Abigail Southers, Krzysztof Kuś, Niek Schoemaker, Esra Balıkçı, Charles Hewes, Marjorie Fournier, Ebru Aydin, Sarah Sayed Hassanein, Luke Slade, Cornelia Kilchert, Jonathan M. Grimes, Lidia Vasiljeva

**Affiliations:** 1 Department of Biochemistry, University of Oxford, Oxford, UK; 2 Division of Structural Biology, Wellcome Trust Centre for Human Genetics, University of Oxford, Oxford, UK; 3 Institut für Biochemie, Justus-Liebig-Universität Gießen, Gießen, Germany; 4 Zoology Department, Faculty of Science, Cairo University, Giza, Egypt; 5 School of Life Sciences, University of Sussex, Brighton, UK; 6 Present Address: Division of Chemistry and Chemical Engineering, California Institute of Technology, Pasadena, CA USA

**Keywords:** RNA, Transcription, X-ray crystallography

## Abstract

Phosphorylation plays a central role in coordinating transcription with pre-mRNA maturation, yet the mechanistic understanding of how transcription is regulated by phosphorylation remains limited. The PP1 phosphatase removes Cdk9-dependent phosphorylation of Pol II and Spt5, triggering transcription termination. Here, we demonstrate that PNUTS enhances PP1 activity, whereas WDR82 mediates an interaction between PP1 and the pre-mRNA 3′-end processing machinery subunit Symplekin. We present the structure of a PNUTS-WDR82-Symplekin-Ssu72 complex, revealing that PNUTS and Symplekin bind WDR82 through distinct interfaces using conserved short linear motifs. Mutations that inactivate PP1’s catalytic activity or disrupt its interaction with either PNUTS or the 3′-end processing machinery led to impaired transcription elongation, inefficient cleavage of nascent transcripts, altered poly(A) site selection, and widespread transcription termination defects. Our findings demonstrate that the PP1 complex is essential for coupling transcription to pre-mRNA 3′-end maturation, ensuring production of functional mRNA.

## Abstract (web summary)

Phosphorylation links transcription to pre-mRNA maturation, but how remains unclear. Here, the authors show that the PP1 phosphatase, guided by PNUTS and WDR82, uses Symplekin to connect the 3′-end processing machinery to Pol II, coupling transcription termination with mRNA cleavage.

## Introduction

Transcription of protein-coding (pc) genes by RNA Polymerase II (Pol II) is a tightly regulated process that is divided into three stages: initiation, elongation, and termination. Eukaryotic mRNA precursors (pre-mRNAs) must undergo extensive processing, including 5′-capping, splicing, 3′-end cleavage and polyadenylation, which are essential to produce a functional mRNA. Coupling of mRNA processing to transcription controls the accuracy, efficiency and order of these reactions. Dynamic phosphorylation of the intrinsically disordered carboxy-terminal domain (CTD) of Pol II and of the conserved transcription elongation factor Spt5 plays an important role in co-transcriptional RNA processing^1–4^. Pol II CTD is composed of the Y_1_S_2_P_3_T_4_S_5_P_6_S_7_ heptad repeats, within which Tyrosine 1 (Tyr1), Serine 2 (Ser2), Threonine 4 (Thr4), Serine 5 (Ser5) and Serine 7 (Ser7) are reversibly phosphorylated during the transcription cycle^5,6^. Kinases and phosphatases coordinate the addition and removal of phosphate groups, generating phosphorylation patterns characteristic of each transcriptional stage, which enable the temporal recruitment of RNA processing factors.

Spt5, together with Spt4, forms the DSIF complex (Supplementary Table 1), which is recruited to Pol II during early elongation and remains associated with Pol II throughout the transcription cycle^7–9^. The Cdk9 kinase of the P-TEFb complex phosphorylates Spt5, Ser2 of the Pol II CTD, and the Negative Elongation Factor, releasing Pol II from promoter-proximal pausing into elongation. Spt5 phosphorylation is conserved and essential for Pol II processivity, whereas loss of Spt5 phosphorylation impairs elongation and leads to termination of transcription^10–13^. It was demonstrated that phosphorylated Spt5 also recruits Rtf1, a subunit of the elongation factor PAF1^14–17^, yet the precise mechanistic contribution of Spt5 phosphorylation to transcription remains unclear.

In eukaryotes, Pol II dislodgement and transcription termination are mechanistically linked to the endonucleolytic cleavage and 3′-end polyadenylation of nascent RNA by the multi-subunit Cleavage and PolyAdenylation machinery (CPA)^18–21^. In yeast, the CPA comprises the Cleavage and Polyadenylation Factor (CPF), the Cleavage Factor IA, and the Cleavage Factor IB^22^. The CPA recognises the AAUAAA element of the polyadenylation signal (PAS), and the CPA endonuclease (Ysh1/CPSF73) cleaves the nascent RNA^22,23^, generating a 5′-monophosphorylated end that is targeted by the exoribonuclease Xrn2, driving Pol II dislodgement from DNA^24,25^. Accurate PAS selection determines the cleavage position, 3′UTR length and composition, features that affect mRNA localisation, translation efficiency and stability^23,26–28^. Premature CPA recruitment also attenuates the expression of selected pc genes^29–32^. The mechanisms underlying PAS selection by the CPA remain poorly understood.

The dephosphorylation of Spt5 by PP1 plays an evolutionarily conserved role in Pol II transcription termination^13,33–36^. Removal of phosphorylation is associated with deceleration of Pol II downstream of the PAS, which was proposed to provide a kinetic advantage to Xrn2 as it degrades nascent RNA to dislodge Pol II^36^. However, recent work has demonstrated that Xrn2 forms a stable complex with Pol II and requires Spt5 to enhance its nucleolytic activity, which is essential for Pol II termination^37^. These findings suggest that Spt5 dephosphorylation could contribute to other aspects of this process, such as PAS choice and/or cleavage efficiency of the nascent transcript by the CPA.

The PP1 phosphatase also dephosphorylates the Pol II CTD. In fission yeast (*Schizosaccharomyces pombe*, *S. pombe*) and budding yeast (*Saccharomyces cerevisiae*, *S. cerevisiae*), PP1 removes phosphate groups from Thr4 and Tyr1, whereas in mammalian cells, it primarily targets phosphorylated Ser5^33,34,38–41^. However, how non-specific phosphatases such as PP1 are recruited and directed during transcription remains poorly understood. Physiologically relevant PP1 holoenzymes form through the association of catalytic and regulatory subunits^42–47^. Different regulatory subunits assemble into PP1 complexes with distinct substrate specificities and function in various cellular pathways^46,48–50^. PP1 interacts with its regulatory subunits through short linear motifs (SLiMs) that dock to specific surface grooves on the catalytic subunit, with the most prevalent being the hydrophobic RVxF motif^42,43,51,52^. The PP1-Nuclear-Targeting Subunit (PNUTS) and the WD40-repeat protein WDR82 interact with and have been proposed to mediate the function of PP1 in transcription (Supplementary Data 1)^36,38,53^. Depletion of PNUTS and WDR82 in mammalian cells leads to failed transcription termination, resulting in readthrough at the ends of transcription units^36,38,54^. More recently, depletion of PNUTS and WDR82 has been shown to affect Pol II release from promoter-proximal pause and premature transcription termination at promoters and enhancers^40,41,54,55^.

PP1 and its putative regulatory subunits have been co-purified with the CPA in yeast^33,56–58^. In budding yeast, PP1 (Glc7) forms part of the APT complex (Associated with Pta1) together with Ref2 (PNUTS), Swd2 (WDR82), the CPA scaffolding subunit Pta1/Symplekin and the Ser5 phosphatase Ssu72^59–63^. The APT complex has been functionally linked to the cleavage-independent 3′-end formation and transcription termination of non-coding (nc)RNAs, as well as suppression of cryptic transcription together with the trimeric Nrd1-Nab3-Sen1 termination complex (NNS)^64–68^. In metazoans, a functionally analogous pathway relies on the Restrictor complex, composed of the arginine-rich zinc-finger protein ZC3H4 and WDR82^55,69^, which cooperates with PP1-PNUTS to suppress cryptic transcription and promote premature termination (Supplementary Data 1)^55,69–73^. Despite the conserved role of PP1 in transcription, how PP1 holoenzymes are organised and activated on chromatin to regulate this process remains unclear.

Our study uncovers a key role for the conserved PP1-PNUTS-WDR82 (PPW) complex in coupling Pol II transcription to pre-mRNA 3′-end maturation. Here, using *S. pombe* as a model, we reconstitute a PPW complex and show that PNUTS stimulates PP1 activity towards Spt5, whereas WDR82 connects the phosphatase to the 3′-end processing machinery through the CPA subunit Symplekin. We delineate the molecular principles that regulate PP1 activity, assembly of the functional complex and its co-transcriptional tethering. Our nascent transcriptome analyses reveal that loss of PP1 activity, disruption of its interaction with PNUTS, or perturbation of PP1 holoenzyme tethering to Symplekin leads to altered PAS choice, defective 3′-end cleavage, transcriptional readthrough and impaired elongation. We propose that multivalent interactions mediated by WDR82 constitute a combinatorial code that enables crosstalk among multiple complexes to regulate transcription.

## Results

### PNUTS/Ppn1 and WDR82/Swd2.2 form a stable complex with PP1/Dis2

**Fig. 1:**
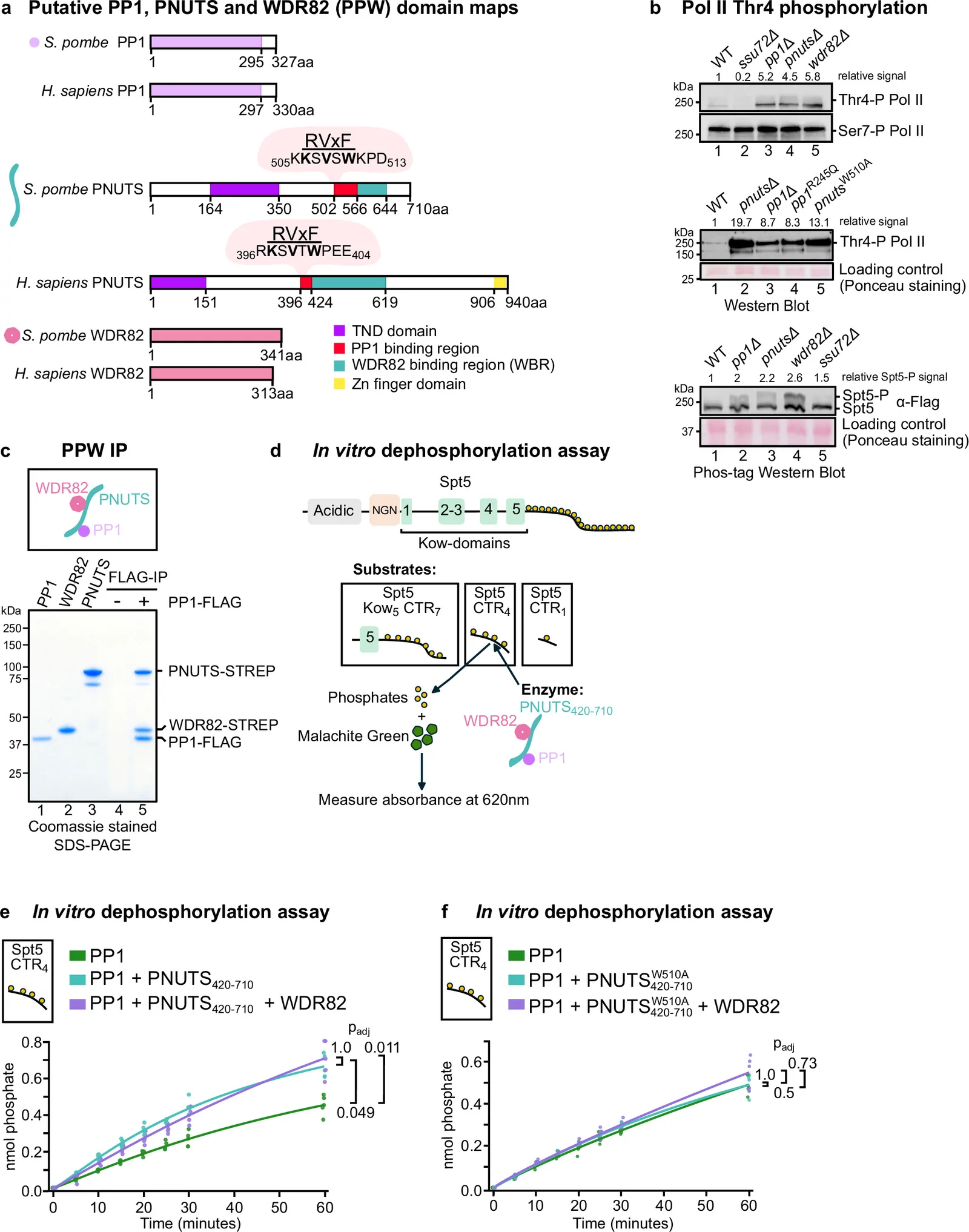
PNUTS stimulates PP1-mediated dephosphorylation of Spt5. **a** Domain maps of PP1, PNUTS and WDR82 from *S. pombe* and *H. sapiens*. TND TFIIS-N-terminal domain. **b** Analysis of the levels of Thr4-P Pol II and Spt5 phosphorylation in *pp1*Δ*, pnuts*Δ*, wdr82*Δ*, pp1*^R245Q^ and *pnuts*^W510A^, WT and *ssu72*Δ strains by Western blotting probed by anti-CTD Ser7-P, Thr4 (repeated twice), FLAG (Spt5 and Spt5-P resolved by the Phos-tag gel, repeated 3 times) and β-actin antibody. Ponceau-stained membrane is shown as loading control. Signal was quantified using Fiji software and presented relative to WT. **c** Reconstitution of the PPW trimeric complex. Purified PP1-FLAG, WDR82 and PNUTS were loaded as inputs. WDR82 and PNUTS co-immunoprecipitated with PP1-FLAG on M2 beads. Co-IP was performed once. Beads were incubated with WDR82-PNUTS as a negative control. **d** Schematic depiction of the phosphatase assay and overview of the Spt5 truncation construct and phosphorylated peptides used as substrates. **e** Phosphatase assays were performed using PP1 alone and in the presence of PNUTS and WDR82, using a 4× phospho-peptide substrate (Spt5-CTR_4_). PNUTS but not WDR82 enhances dephosphorylation of Spt5-CTR_4_ (*n* = 5). Statistical significance was assessed by Kruskal–Wallis test at the 60 min time point, followed by Dunn’s post hoc test with Bonferroni correction. Adjusted *p*-values (*p*_adj_) for all three pairwise comparisons are shown. **f** ${{\mbox{PNUTS}}}_{420-710}^{{\mbox{W}}510{\mbox{A}}}$ cannot enhance PP1 activity. Statistical analysis was performed as described in panel (**e**). Source data for (**b**, **c**, **e**, **f**) are provided as a Source Data file.

PNUTS and WDR82 are conserved across eukaryotes, with the fission yeast orthologues Ppn1 and Swd2.2 showing slightly higher identity to their human counterparts (global alignment 12.3 and 33.2%, respectively) than the corresponding budding yeast proteins (10.9 and 31.9%) (Fig. 1a). The genetic tractability of fission yeast makes it an ideal organism to explore the mechanistic role of PP1 and its regulatory subunits in transcription. For simplicity, we refer to the *S. pombe* orthologues by the metazoan names in this study. Like mammalian PNUTS, the *S. pombe* orthologue is exclusively nuclear (Supplementary Fig. 1A). Secondary structure predictions suggest that PNUTS is largely unstructured, except for its N-terminal region, which adopts a HEAT domain followed by a TFIIS N-terminal-domain-like (TND-like) fold. The remaining disordered regions contain several linear motifs, including a predicted PP1-interacting RVxF motif located at residues 506–510 of *S. pombe* PNUTS (Fig. 1a).

To explore whether PNUTS and WDR82 are required for PP1/Dis2 activity, we assessed the phosphorylation status of known PP1 substrates in whole-cell extracts^33^. This analysis revealed that loss of either PNUTS or WDR82 leads to an accumulation of hyper-phosphorylated Spt5 (Spt5-P) and Thr4-P Pol II (Fig. 1b)^33,74–76^. Levels of Thr4-P Pol II in *pnuts* and *wdr82* deletion strains were similar to levels observed in the strain lacking active PP1 (*pp1*^R245Q^ mutant) (Fig. 1b, lane 4, second panel). No increase in Thr4-P was observed upon loss of Ssu72, consistent with its role as a Pol II Ser5-P specific phosphatase^61,62^ (Fig. 1b, lane 2, top panel). These results suggest that PP1 relies on PNUTS and WDR82 for the dephosphorylation of its substrates during Pol II transcription. Consistent with this hypothesis, mutating the tryptophan residue to an alanine in the _506_KSVSW_510_ portion of the PNUTS RVxF motif predicted to abolish PNUTS-PP1 interaction (*pnuts*^W510A^) leads to increased accumulation of Thr4-P Pol II (Fig. 1b, middle panel lane 5), further supporting the importance of the PNUTS-PP1 interaction in dephosphorylating PP1 substrates during Pol II transcription.

To further understand the roles of PNUTS and WDR82 in PP1-mediated dephosphorylation, we undertook a reductionist in vitro approach using purified proteins. We tested whether PP1, PNUTS and WDR82 can form a complex by incubating individually purified proteins (Fig. 1c, lanes 2 and 3). SDS-PAGE analyses revealed the presence of PNUTS and WDR82 in PP1-FLAG elution, whereas no proteins were recovered from control IP lacking PP1-FLAG (Fig. 1c, lanes 4 and 5). We conclude that the PPW complex is evolutionarily conserved^40,41,53^.

### PNUTS stimulates PP1 activity against Spt5-CTR

To gain insight into how PNUTS and WDR82 contribute to PP1 activity, we established an in vitro dephosphorylation assay (Fig. 1d). The N-terminal part of PNUTS has been shown to be dispensable for interactions with PP1 and WDR82^40,53^; therefore, we expressed and purified recombinant PNUTS_420–710_ harbouring the PP1-interacting RVxF motif and a region predicted to interact with WDR82^53,56^ (Fig. 1a and Supplementary Fig. 1B). To determine the contribution of the PPW subunits to PP1 activity, we tested its activity in vitro against various Spt5 substrates. A phosphorylated Spt5-Kow_5_ construct containing seven CTR consensus repeats (Supplementary Fig. 1C) and synthetic one- or four-repeat phospho-Spt5 peptides were used as substrates (Supplementary Fig. 1D). Phosphatase activity was assessed in the presence or absence of PNUTS and WDR82 and then quantified by measuring phosphate release over time (Fig. 1d). The addition of PNUTS stimulated PP1 activity 1.5-fold for all the substrates (Fig. 1e and Supplementary Fig. 1E, F). In contrast, the PNUTS^W510A^ mutant failed to stimulate PP1 (Fig. 1f). The addition of WDR82 did not stimulate PP1 activity with or without functional PNUTS present (Fig. 1e, f). Together, these results indicate that PNUTS enhances PP1 phosphatase activity towards Spt5 CTR, whereas WDR82 does not contribute to PP1 activity. One repeat peptide seems sufficient for the enzyme to engage with the substrate, as PP1 activity was comparable for all the substrates tested.

### Loss of PP1 activity or regulatory subunits leads to altered pre-mRNA 3′-end processing and defective transcription termination

To investigate the role of PPW complex in transcription, we employed mutant strains to dissect regulatory and catalytic contributions of subunits in vivo: *pnuts*^W510A^, where PNUTS cannot bind and activate PP1 (Fig. 1a and f), alongside a catalytically inactive *pp1*^R245Q^ mutant^77^ (Fig. 1b).

**Fig. 2:**
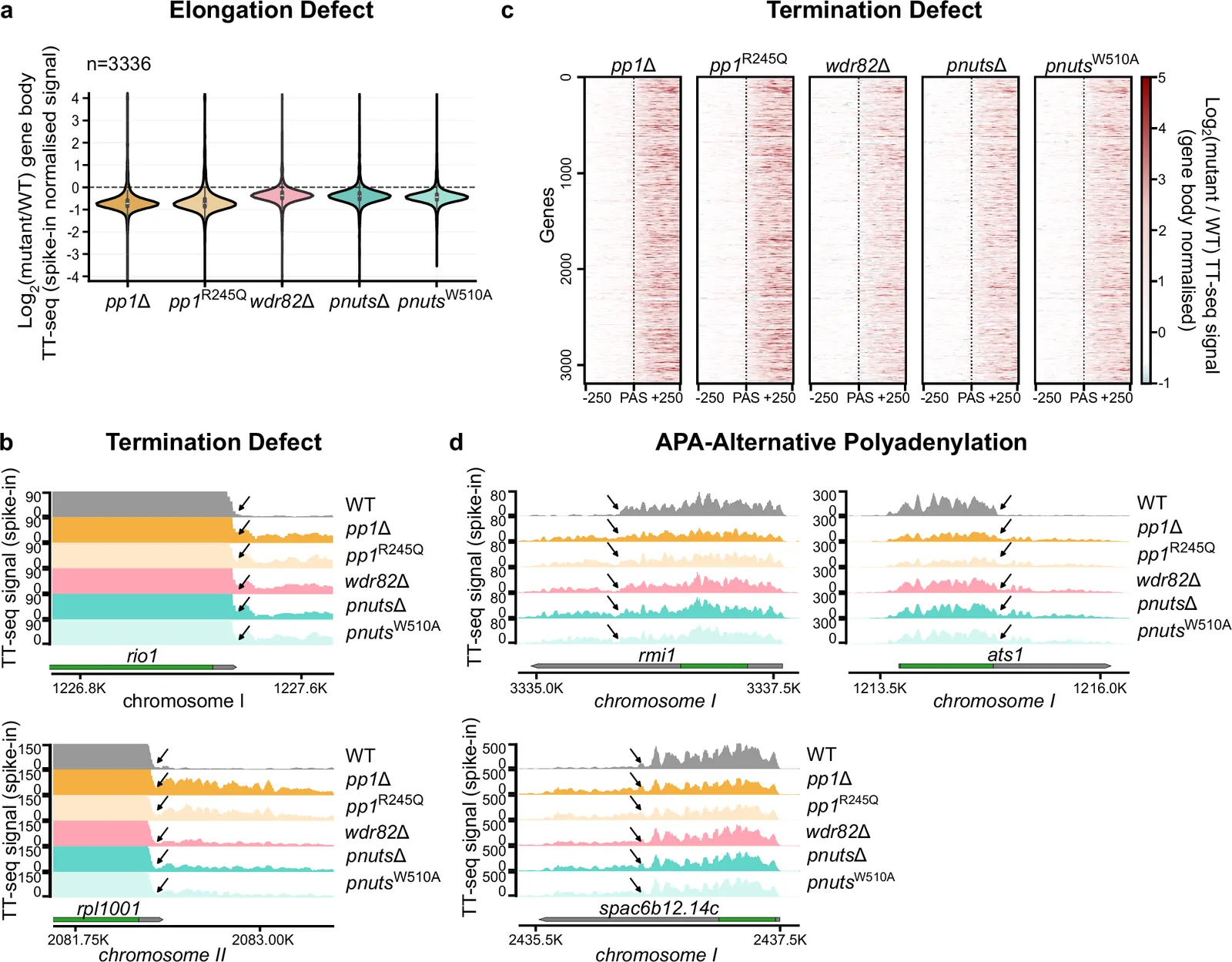
Altered PPW function impairs transcription termination and 3′-end processing. **a** Relative changes in nascent transcription in *pp1*Δ*, pnuts*Δ*, wdr82*Δ*, pp1*^R245Q^ and *pnuts*^W510A^ strains compared to WT. Expression levels across pc genes (*n* = 3336) (selected genes without TUs on the same strand within 250 nt upstream of the TSS or 250 nt downstream of the PAS) were measured by the TT-seq spike-in, normalised, and referenced to the WT strain. Values are the mean of two biological replicates (*n* = 2), which gave equivalent results. Each violin shows the full kernel density of the distribution, with a box plot nested inside it: the centre dot is the median (50^th^ percentile), the bounds of the box are the 25^th^ and 75^th^ percentiles (Q1 and Q3), and the whiskers represent 1.5 × IQR (interquartile range). Points beyond the whiskers, including the minima and maxima, are not drawn individually; they are represented by the surrounding violin density. For display the violins are truncated at ± 4 log_2_ units (3–13 genes per strain lie outside this range), which does not affect the quartiles or whiskers. **b** Transcriptional readthrough, which is a potential hallmark of transcription termination defects, for representative genes in mutants described in (**a**). **c** Heatmaps representing termination defects (readthrough), scaled to expression in the gene body (GB). The defects are global and affect the vast majority of pc genes. **d** Mutants described in (**a**) show changes in 3′UTR usage. Arrows highlight proximal PAS used predominantly in WT cells.

To directly monitor nascent transcription, we performed spike-in normalised transient transcriptome sequencing (TT-seq) in *pp1*∆, *pp1*^R245Q^*, wdr82*∆, *pnuts*∆ and *pnuts*^W510A^ strains. TT-seq revealed a genome-wide elongation defect in all PPW mutants, leading to reduced expression of pc genes (Fig. 2a and Supplementary Fig. 2A). While ncRNAs transcribed by Pol II were less affected, ribosomal RNA genes showed modest downregulation specifically in *pp1*∆ and *pp1*^R245Q^ strains (Supplementary Fig. 2B). All mutants exhibited elevated reads downstream of annotated PAS, as demonstrated at individual gene loci (Fig. 2b) and in genome-wide analyses normalised to gene expression (Fig. 2c), indicating defective transcription termination. The PPW mutants also exhibited a 3′-end processing defect with many genes shifting to more distal PAS usage (Fig. 2d). Increased read density across PAS could result from either altered PAS selection (APA) or reduced CPSF73/Ysh1 activity. We next examined genes with 3′UTRs ≥ 50 nt and identified those with ≥1.5-fold increase in 3′ reads, indicative of possible distal PAS usage, relative to WT in at least two mutants, revealing 514 genes with longer 3′UTRs (3419 unchanged genes) (Supplementary Fig. 2C). The upregulated 3′UTRs observed in PPW mutants exhibited a distinctive bimodal GC content distribution with a second peak at higher GC content (Supplementary Fig. 2D). Furthermore, hexamer analysis revealed enrichment of CG-rich motifs in genes with a shift in 3′UTR usage (Supplementary Fig. 2E), suggesting that the dependency of 3′-end processing on PPW may be modulated by 3′UTR sequence context, which could either contribute to the affinity of CPA binding to nascent RNA, affect Pol II speed or pausing frequency. The TT-seq data demonstrate that the PPW phosphatase complex is essential for efficient transcription elongation, accurate 3′-end formation, and proper termination. Loss of PP1 catalytic activity or disruption of its regulatory subunits induces genome-wide elongation defects and pervasive readthrough transcription. The PP1 complex acts as a key integrator coupling transcription elongation with RNA 3′-end processing and termination. We propose that the PPW complex may participate in regulating CPA function by either contributing to PAS selection or modulating CPSF73/Ysh1 activity.

### WDR82 links PNUTS-PP1 to the CPA machinery

**Fig. 3:**
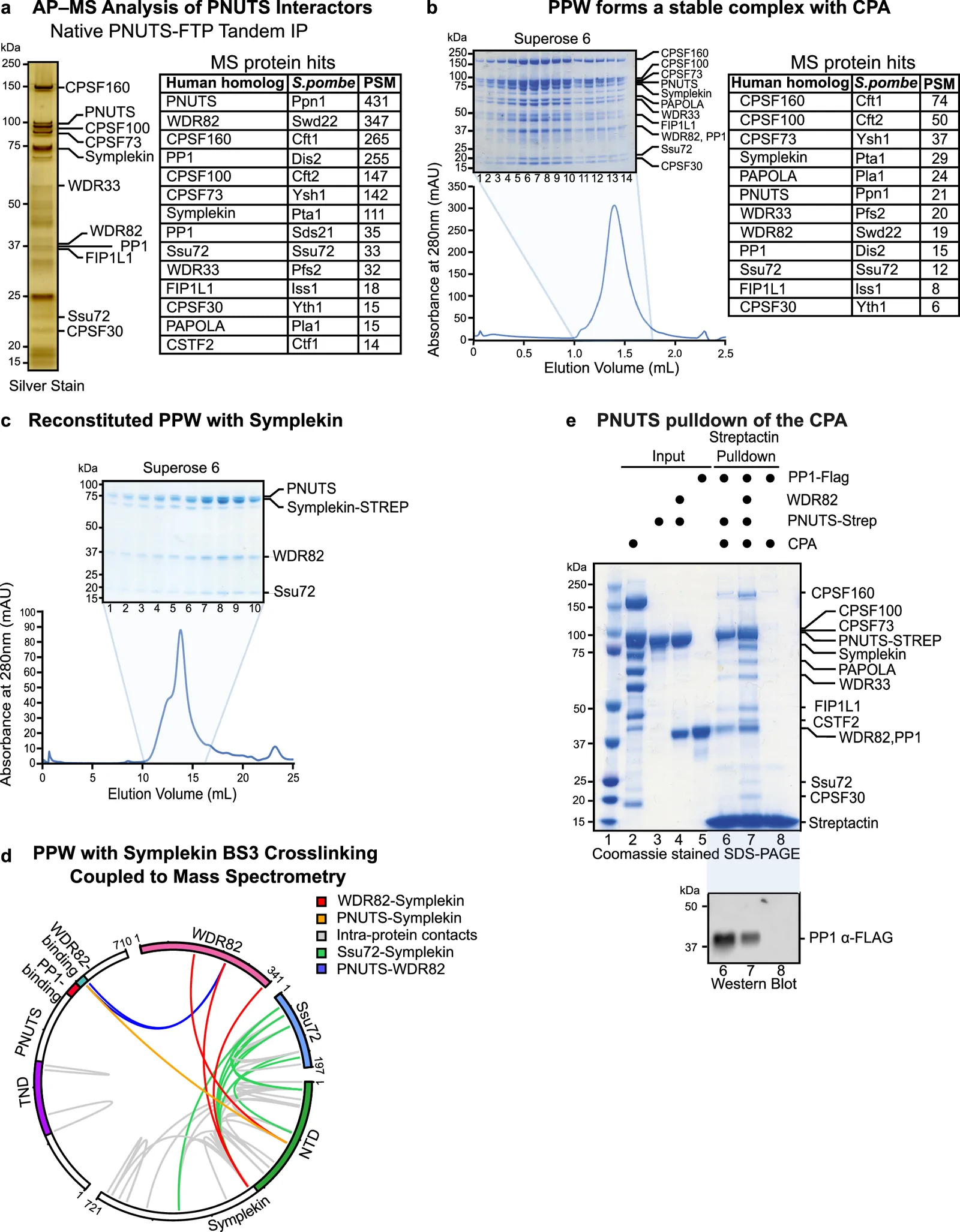
PNUTS and WDR82 assemble with PP1 and the CPA machinery. **a** FTP-tagged PNUTS was purified from *S. pombe*, and proteomic analysis of co-purifying factors identified PP1, WDR82 and multiple components of the CPA machinery (*n* = 2). **b** A stable 12-subunit complex comprising the *S. pombe* CPA machinery and the PP1 holoenzyme was reconstituted in vitro. The table shows the results of the mass spectrometry analyses (*n* = 2). **c** Biochemical reconstitution demonstrates that PNUTS, WDR82, Symplekin and Ssu72 assemble as a stable tetrameric complex (*n* = 1). **d** BS3 cross-linking coupled to mass spectrometry revealed extensive interactions between Ssu72 and Symplekin, primarily within Symplekin residues 1–320. Additional crosslinks were detected among WDR82, the Symplekin_NTD_, and a small region of PNUTS, consistent with biochemical and structural analyses (*n* = 1). **e** WDR82 mediates the interaction of PNUTS with CPA. Coomassie-stained SDS-PAGE and anti-FLAG immunoblotting indicate that PP1 associates with PNUTS independently of WDR82 (lanes 6–7), whereas efficient CPA binding required WDR82 (lane 7) (*n* = 1). No PP1 or CPA were detected in the PNUTS-free control (lane 8). Source data are provided as a Source Data file for (**a**–**c**, **e**).

We next asked how the PPW complex participates in mRNA 3′-end processing. To gain insight into PPW function, we purified *S. pombe* FTP-tagged PNUTS using tandem affinity chromatography and identified proteins co-purified with PNUTS by mass spectrometry (Fig. 3a). Consistent with previously published work, the PPW complex co-purifies with the CPA machinery (Fig. 3a)^58^. In *S. cerevisiae*, the PPW complex interacts with additional subunits Syc1 and Pti1 and forms a complex with Pta1 (Symplekin) and Ssu72, which was termed the Phosphatase Module within the CPF^78,79^. Although it is not fully clear how PPW integrates within *S. cerevisiae* CPF, noncovalent nanoelectrospray ionisation mass spectrometry (nanoESI-MS) combined with computational analysis suggested that the PPW complex interacts with Syc1 and Pti1, which could tether PPW to the CPF via Symplekin^79^. We hypothesised that PPW forms a complex with the 3′-end processing machinery and showed that a stable 12-subunit complex between the *S. pombe* CPF and the PP1 holoenzyme can be reconstituted in vitro using a baculovirus expression system (Fig. 3b). This indicates that the PP1 holoenzyme is part of the CPF, which may explain why defective 3′-end processing is observed by TT-seq upon loss of PPW subunits (Fig. 2d and Supplementary Fig. 2C–E).

Since Symplekin interacts with and activates Pol II Ser5-P phosphatase Ssu72 in humans^63^, forming part of the Phosphatase module in *S. cerevisiae* CPF^78,79^, we hypothesised that PPW interacts with the CPA via Symplekin. To further elucidate how PPW interacts with CPF, we cloned PPW-Symplekin-Ssu72 into a baculovirus construct and attempted to purify the heteropentameric complex using a twin-StrepII tag on Symplekin. While mass spectrometry confirmed the presence of all five subunits in the resulting complex, PP1 was markedly sub-stoichiometric, which we attribute to inefficient expression and folding in insect cells (Fig. 3c). Our data indicate that Symplekin and Ssu72 can form a heterotetrameric complex with PNUTS and WDR82 within CPF. To probe interactions within this PNUTS-WDR82-Symplekin-Ssu72 complex, we employed amine-reactive bis(sulfosuccinimidyl) suberate (BS3) cross-linking coupled to mass spectrometry (XL-MS). We observed numerous crosslinks between Ssu72 and Symplekin, most of which were localised between residues 1 and 320 of Symplekin, consistent with reported structures of Symplekin-Ssu72 complexes from humans^63^ (Fig. 3d). We also observed crosslinks between WDR82, Symplekin_NTD_, and a small region within PNUTS between residues 565 and 591 (Fig. 3d). We conclude that in addition to binding Ssu72, Symplekin_NTD_ directly interacts with WDR82 and PNUTS, and may link the PPW complex to the CPA machinery.

To test this hypothesis directly, we performed in vitro reconstitution experiments. We reconstituted a variant of *S. pombe* CPF lacking PNUTS, WDR82 and PP1, which we term core CPF, by co-expressing the remaining 10 subunits in two MultiBac constructs in Sf9 cells. Full-length Strep-tagged PNUTS was immobilised and incubated with PP1 and the core CPF in the presence or absence of WDR82. The bead-bound fractions were analysed by Coomassie-stained SDS-PAGE and Western blotting with anti-FLAG antibody to allow unambiguous detection of FLAG-tagged PP1 (Fig. 3e). While PP1 bound to PNUTS independently of WDR82 (Fig. 3e, lanes 6 and 7), the CPF core associated with PNUTS and PP1 only in the presence of WDR82 (Fig. 3e, lane 7), suggesting that WDR82 plays a central role in bridging the PPW complex and the CPA machinery.

### Structural insight into PPW assembly with the 3′-end processing machinery

**Fig. 4:**
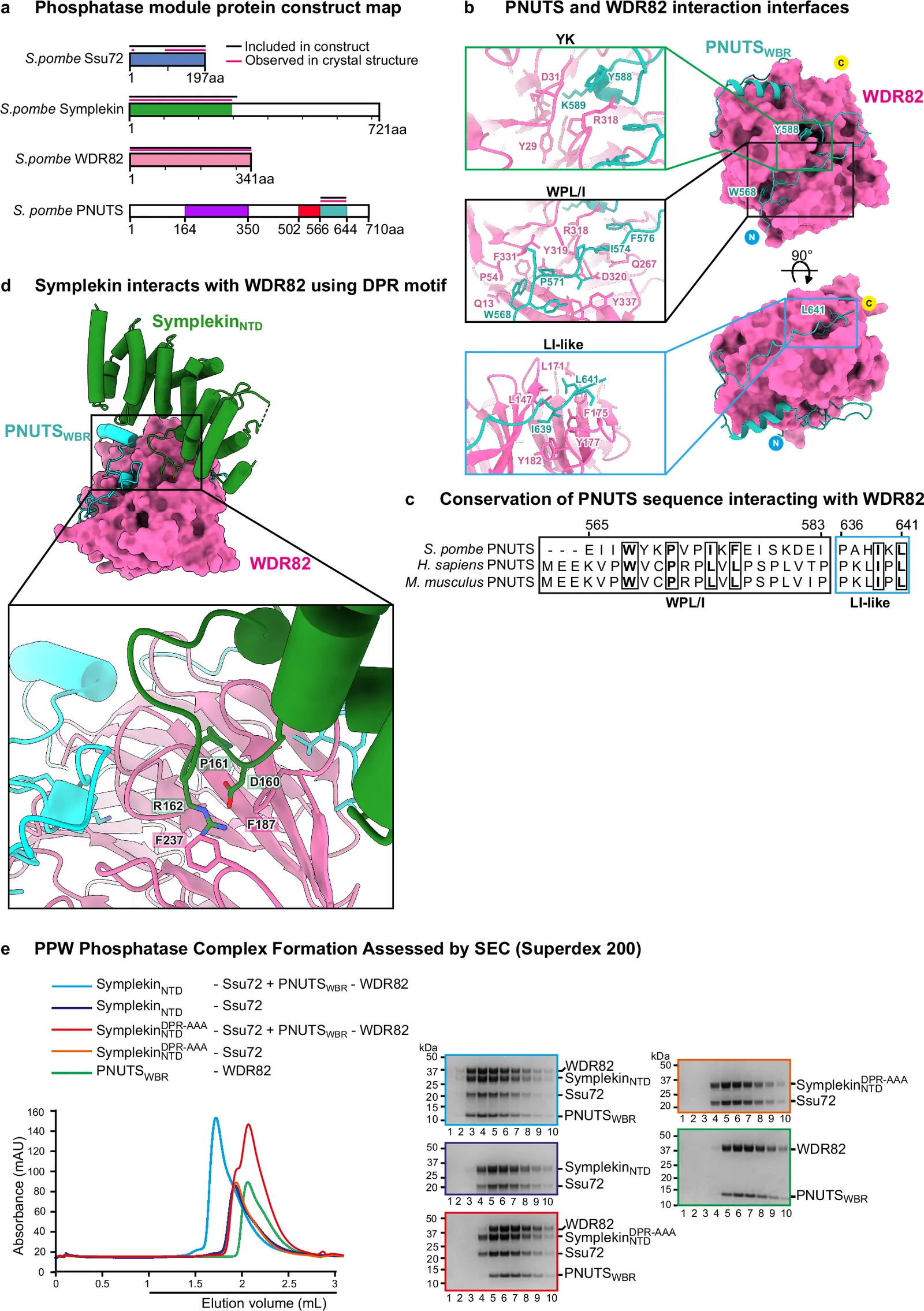
WDR82 engages PNUTS and recruits Symplekin via its conserved DPR motif. **a** Domain maps of Symplekin, PNUTS, Ssu72 and WDR82 from *S. pombe*. Regions included in the X-ray crystallography constructs are indicated via a black line, and the portions resolved in the crystal structure are highlighted via a pink line. **b** The structure of the PNUTS_WBR_-WDR82 complex was solved by X-ray crystallography. PNUTS_WBR_ makes extensive hydrophobic contacts with WDR82, mediated by conserved WPL and LI motifs, along with additional helix-groove interactions. **c** Conservation of PNUTS-WDR82 interacting motifs (WPL/I and LI-like motifs). **d** Microseeded crystallisation yielded a 3.8 Å structure of the reconstituted PNUTS_WBR_-WDR82-Symplekin_NTD_-Ssu72 complex. Symplekin_NTD_ forms a HEAT-repeat domain that contacts WDR82 through a conserved DPR motif in its extended α7-8 loop, and PNUTS provides additional stabilising contacts. **e** Analyses of the complex for PNUTS_WBR_-WDR82 with Symplekin_NTD_-Ssu72 or ${{{\rm{Symplekin}}}}_{{{\rm{NTD}}}}^{{{\rm{DPR}}}-{{\rm{AAA}}}}$-Ssu72 formation by analytical SEC (Superdex 200). ${{{\rm{Symplekin}}}}_{{{\rm{NTD}}}}^{{{\rm{DPR}}}-{{\rm{AAA}}}}$ mutant formed a heterodimer with Ssu72 but failed to assemble with PNUTS_WBR_ -WDR82 into the hetero-tetramer, in contrast to wild-type Symplekin_NTD_-Ssu72. Source data are provided as a Source Data file.

We next sought to understand how WDR82 recruits PNUTS and PP1 to the CPF via Symplekin. Our in vitro reconstitution and crosslinking data show that WDR82 directly interacts with PNUTS and the N-terminal domain of Symplekin. Furthermore, PNUTS and WDR82 form a stable heterodimer (Supplementary Fig. 3A). Our data support a model where WDR82 bridges PP1-PNUTS to the CPA. We used our XL-MS data together with AlphaFold2 to define the WDR82-binding region of PNUTS (PNUTS_WBR_), which we predict to encompass PNUTS residues 565–644 (Fig. 4a). We confirmed this prediction by expressing PNUTS_WBR_-WDR82 and determining its structure by X-ray crystallography to 1.6 Å resolution. All residues of PNUTS_WBR_ and all but two of the final residues of WDR82 were successfully modelled (Fig. 4b). PNUTS_WBR_ interacts extensively with WDR82, burying a surface area of ~3500 Å^2^ (Fig. 4b). Most interactions between PNUTS_WBR_ and WDR82 are hydrophobic and ionic. A stretch of hydrophobic residues _565_EIIWYKPVPIKF_576_ (WPL/I) makes contacts along a groove in WDR82, whilst another stretch of hydrophobic interactions is mediated by residues 639-641 of PNUTS, which we refer to as the LI-like motif (Fig. 4b, c). Both the WPL/I and LI-like motifs are highly conserved (Fig. 4c). The region between 586 and 590 (_586_RGYKC_590_) forms a half-helix over the centre of the WD40 domain of WDR82, inserting K_589_ into the central pore. Additionally, PNUTS _599_PEATSEIEREKN_610_ forms a helix enriched in charged residues, providing another interaction site.

We then reconstituted a PNUTS_WBR_-WDR82-Symplekin_NTD_-Ssu72 complex by mixing individually purified PNUTS_WBR_-WDR82 and Symplekin_NTD_-Ssu72 complexes, followed by size-exclusion chromatography (Supplementary Fig. 3B), and crystallised the resulting complex, which diffracted to 3.8 Å resolution. We used the experimentally determined PNUTS_WBR_-WDR82 crystal structure, as well as AlphaFold2-generated models of Symplekin_NTD_ and Ssu72, as initial search models to solve the tetramer structure by molecular replacement. The density for Ssu72 is poor and is only visible for most of the low-molecular-weight protein tyrosine phosphatase (LMWPTP) fold, which constitutes the core of Ssu72. The density for the cap domain, as well as a helix and a β-strand from the LMWPTP were absent from the structure (Supplementary Fig. 3C, D). The portion of Ssu72 that could be modelled includes the interface with Symplekin_NTD_, and the critical residues reported for the human Symplekin_NTD_-Ssu72 interface are highly conserved (Supplementary Fig. 3E). While we do not fully understand the reason for the missing electron density, we speculate that the Ssu72 cap domain is flexible relative to the Symplekin-bound LMWPTP domain and adopts multiple orientations within the crystal. Within the tetrameric complex, the PNUTS_WBR_-WDR82 heterodimer maintains the same overall conformation as observed when the PNUTS_WBR_-WDR82 structure was solved independently (Supplementary Fig. 3F).

Symplekin_NTD_ is composed of twelve antiparallel helices arranged in a HEAT domain, similar to its metazoan counterpart and contacts both Ssu72 (Supplementary Fig. 3E) and WDR82 (Fig. 4d). The loop connecting α-helices 7 and 8 (hereafter referred to as the α7-8 loop) is long, with the tip of the loop protruding outwards from Symplekin and inserting into a pocket in WDR82 formed by two blades of the WD40 domain (Fig. 4d). The tip of the α7-8 loop is formed by three amino acids: Asp_160_, Pro_161_ and Arg_162_, hereafter referred to as the DPR motif. The model proposes that Proline 161 is in a *cis* configuration and maintains the sidechains of the flanking Asp_160_ and Arg_162_ residues in an outward-facing orientation (Supplementary Fig. 4A). The sidechains of the flanking Asp_160_ and Arg_162_ make electrostatic interactions with each other, further stabilising the orientation of this DPR motif (Supplementary Fig. 4A). The aspartic acid (Asp_160_) and arginine (Arg_162_) residues of the DPR motif form ion-π interactions with Phe_187_ and Phe_237_ of WDR82 (Fig. 4d and Supplementary Fig. 4A).

The DPR motif was previously shown in vivo to be required for the interaction of WDR82 with SET1A and ZC3H4 in mammals as part of the SET1 and Restrictor complexes^54,80^. We therefore hypothesised that the DPR motif is an evolutionarily conserved motif utilised by diverse WDR82-interactors. To test the importance of the Symplekin DPR motif observed in our crystal structure for binding WDR82, we expressed and purified a variant of the Symplekin_NTD_-Ssu72 heterodimer in which the DPR motif was replaced with three alanines. ${{{\rm{Symplekin}}}}_{{{\rm{NTD}}}}^{{{\rm{DPR}}}-{{\rm{AAA}}}}$ was fully functional in the formation of a heterodimer (${{{\rm{Symplekin}}}}_{{{\rm{NTD}}}}^{{{\rm{DPR}}}-{{\rm{AAA}}}}$-Ssu72) with Ssu72 and eluted in the same volume as the Symplekin_NTD_-Ssu72 heterodimer in analytical size exclusion chromatography (Fig. 4e, orange), showing that Symplekin binding to Ssu72 is unaffected by the loss of the DPR motif. Mixing Symplekin_NTD_-Ssu72 and PNUTS_WBR_-WDR82 resulted in the formation of the hetero-tetramer (Fig. 4e, blue), and ${{{\rm{Symplekin}}}}_{{{\rm{NTD}}}}^{{{\rm{DPR}}}-{{\rm{AAA}}}}$-Ssu72 did not interact with PNUTS_WBR_-WDR82 (Fig. 4e, red). This demonstrates that the DPR motif in Symplekin is essential for binding WDR82. Our structure additionally shows limited interaction between PNUTS_WBR_ and Symplekin_NTD_, suggesting that Symplekin and PNUTS bind WDR82 independently. To test this, we repeated our reconstitution experiments without PNUTS and evaluated the results by analytical SEC. Our results showed that Symplekin_NTD_-Ssu72 bound WDR82, whereas ${{{\rm{Symplekin}}}}_{{{\rm{NTD}}}}^{{{\rm{DPR}}}-{{\rm{AAA}}}}$-Ssu72 did not, confirming that Symplekin and PNUTS bind WDR82 independently (Supplementary Fig. 4B). Moreover, these experiments suggest that PP1 and Ssu72 do not directly interact.

To further delineate the importance of different regions of PNUTS engaged in the interaction with WDR82, we generated a PNUTS construct in which residues 566–591 within the WBR, comprising the highly conserved WPL/I (Fig. 4c) and positive/negative helix, were replaced with a glycine-alanine linker (referred to as PNUTS^WPL-G/A^) (Supplementary Fig. 4C, D). This mutant retained the ability to pull down PP1 but lost the ability to pull down Symplekin_NTD_-Ssu72 even in the presence of WDR82 (Supplementary Fig. 4D, lanes 5–8). This suggests that the WPL/I motif and the helix are essential for the PNUTS-WDR82 interaction, explaining its conservation (Fig. 4c), and this interaction is required to link PNUTS-PP1 with Symplekin-Ssu72.

### The DPR motif is necessary to mediate an interaction between the CPF and PP1 phosphatase complex and is required for PP1 substrate dephosphorylation

**Fig. 5:**
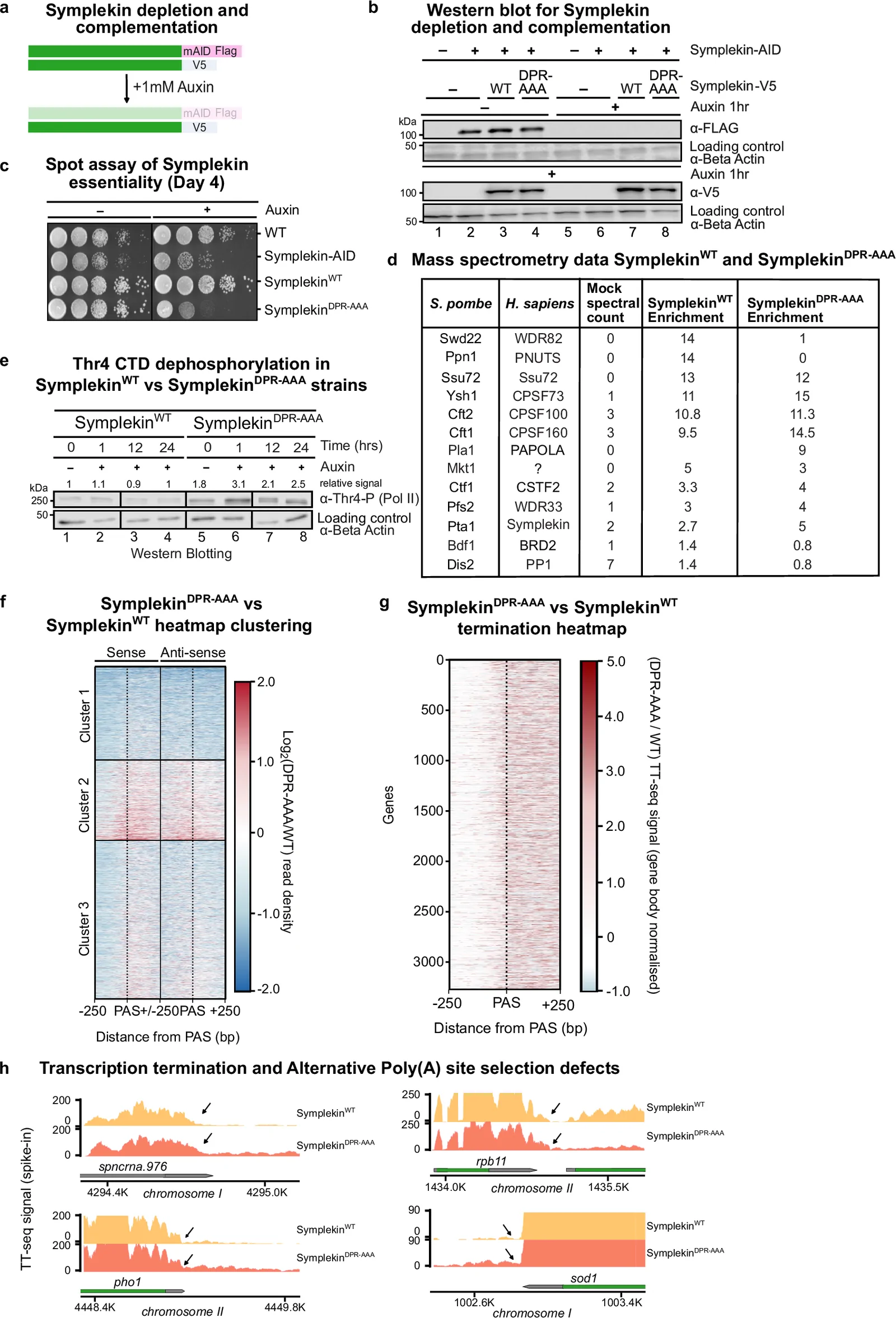
The Symplekin DPR motif is required for CTD dephosphorylation and efficient transcription termination. **a** Schematic of endogenous Symplekin with AID and FLAG-tag, and secondary copies of Symplekin^WT^ and Symplekin^DPR-AAA^ with V5-tag. **b** Western blot analyses of Symplekin^WT^ and Symplekin^DPR-AAA^ with β-actin used as a loading control. The FLAG and V5 WB were repeated twice with similar results. **c** Serial dilutions of WT, Symplekin-AID, Symplekin^DPR-AAA^ and Symplekin^WT^ strains to assess growth. Cells were grown on YES plates at RT in the presence of auxin. **d** Purification of V5-tagged Symplekin^WT^ and Symplekin^DPR-AAA^ followed by proteomic analysis of interacting partners, using mock without V5 tag as a control (*n* = 1). **e** Analyses of Pol II Thr4-P levels in Symplekin^WT^ or Symplekin^DPR-AAA^ strains following depletion of AID-Symplekin (representative data of two repeats). Thr4-P signal was quantified using Fiji software and shown as relative to WT. **f** TT-seq analysis of Symplekin^WT^ and Symplekin^DPR-AAA^ following auxin treatment. Representative heatmap of gene expression +/−250 base pairs centred around the PAS, presented as log_2_ fold ratio relative to Symplekin^WT^. Sense and antisense strands presented as log_2_ fold ratio relative to Symplekin^WT^ and clustered. **g** Heatmap of termination defects (readthrough), scaled by GB expression, presented as log_2_ fold ratios relative to Symplekin^WT^. **h** Representative snapshots of Symplekin^WT^ and Symplekin^DPR-AAA^ TT-signal. Arrows highlight proximal PAS used predominantly in WT cells. Source data are provided as a Source Data file for (**b** and **e**).

To dissect the role of the Symplekin DPR motif in mediating the CPA-PPW interaction, we introduced alanine substitutions at the DPR motif (DPR → AAA) to generate the Symplekin^DPR-AAA^ mutant in vivo. Symplekin is an essential gene (Supplementary Fig. 5A), therefore we introduced an additional copy of the Symplekin gene with the DPR-AAA mutation into a strain in which the endogenous Symplekin gene was tagged with an Auxin-inducible degron (AID) (Fig. 5a). AID-tagged Symplekin was efficiently depleted after growing cells for 1 h (Supplementary Fig. 5B) and remained depleted following 24 h in the presence of 1 mM auxin. Complementing the Symplekin-AID strain with a wild-type copy of Symplekin verified that Symplekin^WT^ and Symplekin^DPR-AAA^ were expressed at similar levels (Fig. 5b). Complementation with Symplekin^WT^ but not Symplekin^DPR-AAA^ rescued growth after auxin-induced depletion of endogenous Symplekin (Fig. 5c), suggesting that the interaction between CPA and PPW mediated by the DPR motif in Symplekin is essential for cell viability.

Analysis of native purifications of V5-tagged Symplekin by mass spectrometry revealed that, in contrast to WT Symplekin, which co-purifies with the entire CPA complex, including PPW, Symplekin^DPR-AAA^ lost the ability to interact with PPW, however interactions with the rest of CPA were retained, demonstrating that the overall integrity of the CPA was not compromised by the DPR-AAA mutation (Fig. 5d). PP1 interacted non-specifically with V5 beads, as evidenced by its presence in the no-tag control (mock). We conclude that the DPR motif of Symplekin is important for interacting with WDR82 and recruitment of PNUTS, WDR82 and PP1 to the CPA machinery.

To determine if the interaction between PPW and the CPA is necessary for the dephosphorylation of PP1 substrates involved in transcription, we assessed phosphorylation of Thr4 of the Pol II CTD upon depletion of the endogenous Symplekin in cells complemented with Symplekin^WT^ or Symplekin^DPR-AAA^. Expression of Symplekin^DPR-AAA^ before depletion of AID-tagged Symplekin^WT^ led to the accumulation of Thr4-P Pol II, suggesting that Symplekin^DPR-AAA^ may have a dominant negative effect (Fig. 5e, compare lanes 5 to 1). Depletion of endogenous AID-tagged Symplekin led to a further increase in the levels of phosphorylated Pol II (Fig. 5e, lane 6), demonstrating that association of the PPW complex with the CPA machinery is important for dephosphorylation of Pol II by PP1.

We demonstrate that WDR82 bridges PNUTS-PP1 to the CPA machinery via Symplekin. Both PNUTS and WDR82 are important for the dephosphorylation of PP1 substrates in vivo, but only PNUTS can directly stimulate PP1 activity. Our structural and biochemical data demonstrate that WDR82 plays a prominent role in mediating the interaction between CPA and PPW complexes by interacting with the DPR motif of Symplekin, while PNUTS mediates the interaction between WDR82 and PP1.

### Loss of the physical link between CPA and the PP1 phosphatase complex leads to altered 3′-end processing and transcription termination defects

We next investigated the global transcriptional consequences of disrupting the interaction between the CPA and PPW complex. We performed spike-in normalised transient transcriptome sequencing (TT-seq) in the Symplekin^WT^ or Symplekin^DPR-AAA^ cells. Analysis of nascent transcription revealed a widespread increase in read density downstream of annotated PAS in the Symplekin^DPR-AAA^ strain compared with Symplekin^WT^. The readthrough past the annotated TES was particularly pronounced for genes in cluster 2 (Fig. 5f), and a mild reduction in TT-seq signal across gene bodies was also observed, which is not due to readthrough transcription since it does not correlate strongly with antisense transcription (Fig. 5f). The post-PAS signal was normalised to expression levels to accurately quantify the termination defects. Normalisation confirmed that the 3′-end processing and termination defects associated with Symplekin^DPR-AAA^ globally affect transcription across the genome (Fig. 5g). For a subset of genes, readthrough also reduced transcription of the neighbouring gene (tandem orientation of the genes). For example, at the *rbp11-spac3a12.08* locus, readthrough from *rbp11* interfered with transcription of the downstream *spac3a12.08* gene, revealing how readthrough transcription could negatively impact gene expression from the adjacent promoter (Fig. 5h). We next compared the readthrough defects in Symplekin^DPR-AAA^ with those in the PPW mutants. Genes with increased levels of 3′-end readthrough, observed in at least two PPW mutants, showed a substantial overlap with genes exhibiting readthrough in the Symplekin^DPR-AAA^ strain (Supplementary Fig. 5C, left). This trend was consistently observed across multiple comparisons (Supplementary Fig. 5C, right).

Alternative PAS usage was observed in Symplekin^DPR-AAA^ for a subset of genes; these alterations did not correlate with gene length and showed only minor differences in GC content relative to unaffected genes (Supplementary Fig. 5D, E). No significant alterations in ncRNAs expression were observed in Symplekin^DPR-AAA^ apart from a slight decrease in rRNA levels (Supplementary Fig. 5F). In summary, we identified a direct functional link between CPA and the PPW complex, which is integral to 3′-end processing and transcription termination.

## Discussion

**Fig. 6:**
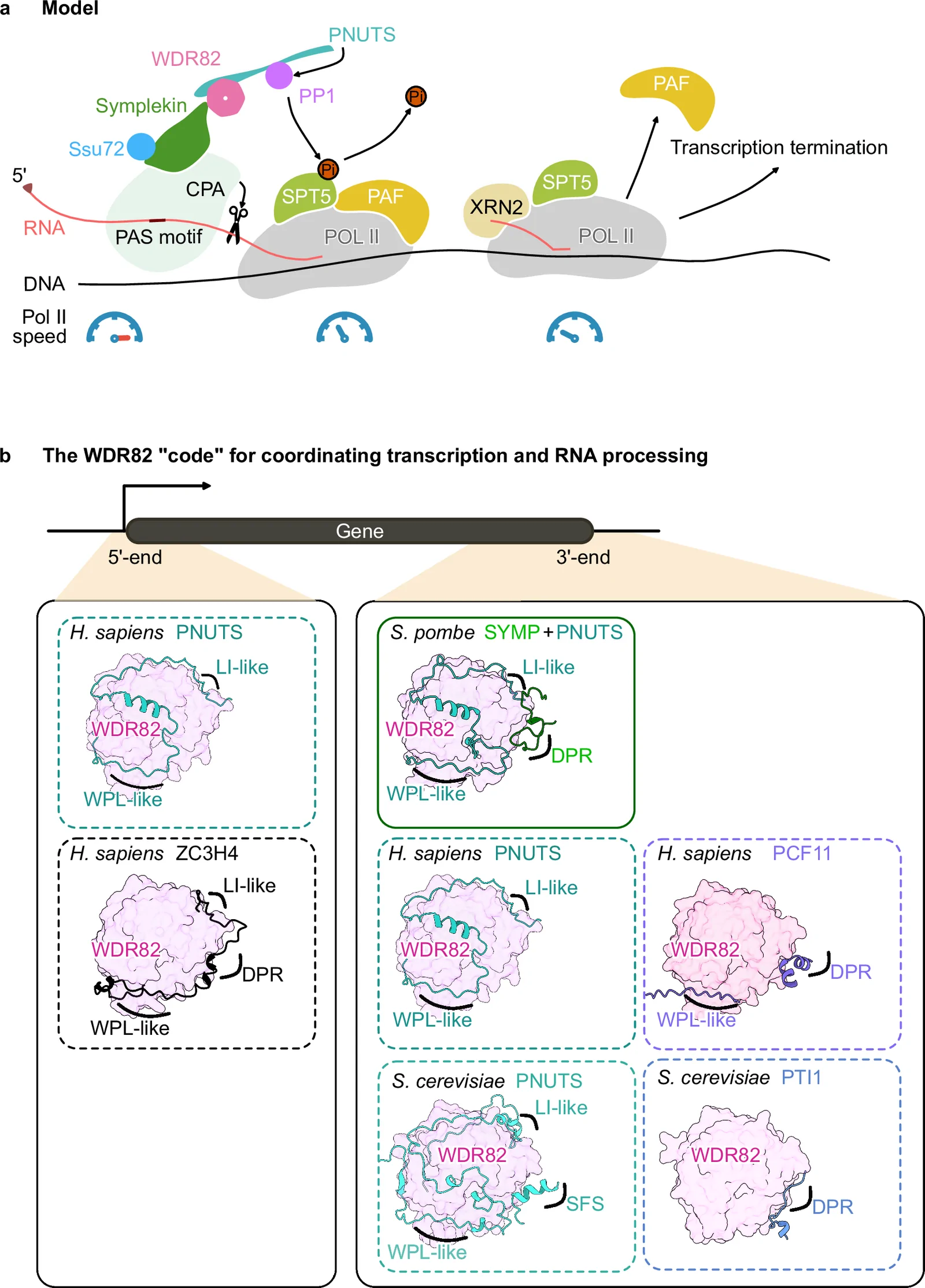
Symplekin recruits PP1 holoenzyme to the CPA machinery, coupling PAS recognition with Spt5 dephosphorylation and transcription termination. **a** Model describing how PAS recognition by the CPA is linked to dephosphorylation of Spt5 via recruitment and activation of PP1 by WDR82 and PNUTS. Symplekin acts as a scaffold that links the phosphatase PPW complex to the 3′-end processing CPA machinery via the DPR motif. WDR82 bridges this interaction, while PNUTS not only recruits PP1 but also enhances its activity toward Spt5. Dephosphorylation of Spt5 can subsequently slow Pol II, promote dissociation of the PAF complex, and facilitate 3′-end processing and transcription termination. **b** WDR82 functions as a central hub that coordinates multiple machineries involved in transcriptional regulation at both the 5′ and 3′-ends of genes. Building upon the structural data presented in this study (solid box), we employed AlphaFold modelling (dashed boxes) to predict how conserved SLiMs could engage with WDR82 to mediate the formation of the diverse set of complexes.

Transcription termination requires the action of phosphatases to remove phosphorylation marks associated with productive elongation from Pol II and transcription factors^81^. The PPW holoenzyme has emerged as a conserved central regulator of the transition from transcription elongation to termination, acting at the 3′-ends of genes and the promoter-proximal region^33–36,38–40,69–72,80,82–86^. By providing insight into how PP1 is recruited and regulated as well as defining the modular interfaces that connect PP1, PNUTS, WDR82 and Symplekin, this work reveals how the conserved PPW complex couples transcription with RNA 3′-end processing. We propose a model in which PP1, activated by PNUTS, is recruited to elongating Pol II via WDR82 and Symplekin, thereby coordinating dephosphorylation of transcription machinery with PAS selection and pre-mRNA 3′-end cleavage during the elongation-termination transition (Fig. 6a). WDR82 acts as a hub that recognises multiple SLiMs in PNUTS and Symplekin via conserved residues (Fig. 4b–d and Supplementary Fig. 6A), creating a platform where the PP1 holoenzyme, the CPA machinery, and Pol II converge at the 3′-ends of genes. Our results revise the prevailing view that PP1 functions primarily downstream of RNA cleavage and instead support a model in which PPW recruitment to the CPA modulates PAS selection and transcription termination.

We propose that the PPW complex coordinates PAS selection and cleavage efficiency through several potential mechanisms. Firstly, PPW association with the CPA can induce conformational changes, thereby influencing PAS recognition or modulating the efficiency of endonucleolytic cleavage. This could be caused by PPW-mediated dephosphorylation of CPA subunits, which may alter their affinity or specificity for PAS elements and/or influence the pre-mRNA cleavage efficiency by CPSF73/Ysh1. PNUTS could facilitate recruitment of CPA to PAS by interacting with nascent RNA via its zinc finger domain, which is present in mammalian PNUTS (Fig. 1a)^87^. Secondly, PPW could facilitate dissociation of elongation factors from Pol II, inducing rearrangements in the elongation complex and slowing Pol II elongation dynamics. PP1-mediated dephosphorylation of Spt5 has been reported to slow Pol II near gene ends, and we propose that this deceleration extends the time window for CPA recognition of the PAS and promotes efficient cleavage. Loss of elongation factors could allosterically destabilise the Pol II-DNA-RNA complex, making Pol II more susceptible to Xrn2 dislodgement, consistent with a unified allosteric-torpedo model^88^. Evidence indicates that PPW promotes transcription termination primarily by remodelling the elongation complex and slowing Pol II, rather than by directly regulating RNA cleavage. PP1, PNUTS and WDR82 can mediate cleavage-independent termination via the NNS complex in budding yeast and the Restrictor complex in metazoans^54,57,59,60,64–66,69,71,89,90^, demonstrating that the PPW complex can drive transcription termination independently of CPA endonucleolytic activity. Consistently, the Restrictor has been proposed to drive transcription termination by slowing Pol II^71^. Additionally, PP1-mediated transcription termination requires dissociation of the PAF complex^17^, which can lead to rearrangements of the Pol II elongation complex and a reduction in Pol II speed^91,92^. Dephosphorylation of Spt5 by PP1 is also associated with the deceleration of Pol II at the 3′-ends of genes, and Pol II speed has been proposed to contribute to the recruitment of RNA processing factors^93–102^.

We present the experimental structure of a PNUTS-WDR82-Symplekin-Ssu72 complex, demonstrating that PNUTS and Symplekin bind to WDR82 via distinct interfaces, each using well-defined SLiMs. We define three conserved WDR82-interacting SLiMs: WPL/I, LI-like and DPR. In our experimental structures, PNUTS utilises WPL/I and LI-like motifs, whereas Symplekin utilises a DPR motif to interact with the PPW complex. We demonstrate that the physical link between PPW and CPA is important for transcription termination and pre-mRNA 3′-end processing. Comparison of Symplekin orthologues across species showed that the DPR motif is present only in fission yeast Symplekin (Supplementary Fig. 6A–D).

While PNUTS, WDR82, and PP1 orthologues have now been identified across a range of eukaryotes, only in yeast species are these proteins stably integrated into the CPA. Metazoan Symplekin lacks a DPR motif, which explains why mammalian PNUTS-WDR82-PP1 does not stably co-purify with CPSF components^22,103^. Despite the absence of the DPR motif in human Symplekin, purification of human 3′-end processing complexes using PAS-containing RNA as a bait revealed the presence of PNUTS and PP1 (but not WDR82) in addition to the CPA components^103^. Reciprocally, the CPA was shown to co-purify with mammalian PNUTS, together with WDR82 and PP1^40^. Together, these data suggest that a functional link between PPW and the CPA is conserved across eukaryotes, albeit potentially mediated through different CPA subunits or alternative interaction surfaces. AlphaFold modelling suggests that these motifs engage the same DPR pocket and WPL groove on WDR82 as observed in the PPW-Symplekin structure (Figs. 6B and 4B–D). WDR82 has been proposed to interact with the CFIIm component hPCF11 to promote premature transcription termination during HIV infection^82^. The WDR82-hPCF11 interface has not been defined experimentally; however, hPCF11 contains both a DPR motif and a WPL-like motif, analogous to those found in PNUTS (Fig. 6b and Supplementary Fig. 7). The position of the DPR motif is not conserved across partners, although the underlying binding principles are maintained. The absence of a DPR motif in budding yeast Symplekin is surprising given that orthologs of PNUTS, WDR82 and PP1 exist as a stable submodule of CPF together with other *S. cerevisiae-*specific subunits, Syc1 and Pti1^78,79^. Due to the highly conserved nature of the DPR-binding pocket, we speculate that the DPR motif resides in another CPA subunit in *S. cerevisiae*. Interestingly, the budding yeast ortholog of PNUTS (Ref2) has WPL/I, LI and SFS (equivalent of DPR) motifs (Fig. 6b). The CPF subunit Pti1 contains a DPR motif and is part of the CPF phosphatase module, therefore Pti1 could be the interaction partner of *S. cerevisiae* WDR82 (Fig. 6b).

Consequently, we propose that while the subunits contributing to the PPW-CPA interface have diverged between species, the overarching principle of PPW engagement with the 3′-end processing machinery/termination complexes is evolutionarily conserved. The binding grooves of these SLiMs on WDR82 are distinct and non-overlapping, suggesting that multiple WDR82 interactors can co-occupy the same WDR82 molecule. We propose a combinatorial WDR82-binding code, but the molecular rules that govern this code remain unknown, especially where a protein contains WPL/I, LI-like and DPR motifs. This is especially pertinent to metazoan WDR82, which is an integral subunit of multiple complexes that regulate transcription in distinct and even antagonistic ways. In addition to the PPW complex, WDR82 also binds ZC3H4 to form the Restrictor complex, which restricts nc transcription in metazoans through a DPR motif in the ZC3H4 subunit^54,69^. The Restrictor was originally proposed to consist solely of ZC3H4 and WDR82, and it was suggested that PNUTS and ZC3H4 binding to WDR82 would be mutually exclusive. AlphaFold predictions of the binary ZC3H4-WDR82 complex show that ZC3H4 forms extensive interactions with WDR82, in addition to the DPR-binding pocket, through the WPL/I and LI binding grooves of WDR82 (Fig. 6b). However, we do not rule out that hybrid complexes could be formed via different Short Linear WDR-Interacting motifs (WDR-SLiMs). AlphaFold predictions of a trimeric PNUTS-WDR82-ZC3H4 complex show PNUTS occupying the WPL/I and LI-like grooves, while ZC3H4 occupies only the DPR-pocket, in agreement with our experimental PNUTS-WDR82-Symplekin-Ssu72 structure (Supplementary Fig. 6E, F). In support of this, ZC3H4 has recently been shown to co-purify with the PPW complex^69^. WDR82 is also part of the H3K4 histone methyltransferase SET1 complex, in *S. cerevisiae* and mammalian cells^80,85,104,105^. WDR82 binding to the SET1 complex (Supplementary Data 1) has been proposed to antagonise Restrictor function in certain loci, through a DPR motif in SET1A. Yet, PNUTS has not been identified in SET1 complexes. Incorporation of WDR82 into the SET1 and 3′-end processing/termination complexes has been proposed to be mutually exclusive, as reported for Restrictor-ZC3H4 in mammalian cells^54^. This may apply to the NNS complex, as WDR82 has been shown to be important for APT recruitment to snoRNA genes that rely on NNS for termination^57,60,85^. However, a physical link between NNS and the APT complex has not yet been reported. These data support the prediction that SET1A occupies all binding surfaces on WDR82 (Supplementary Figs. 6G and 7). These seemingly contradictory observations underscore the need to experimentally validate in silico-predicted structural models. Future structural studies with experimental techniques will be required to validate this combinatorial WDR82-binding code.

This principle could have broader implications beyond the recruitment of the PPW complex to RNA processing machineries. WDR82 was proposed to interact with and mediate the recruitment of the silencing HUSH complex to sites of transcription termination^106^. The HUSH complex has been implicated in the suppression of foreign DNA expression by mediating the recruitment of H3K9 histone methyltransferase. Interestingly, the TASOR subunit of HUSH has a DPR motif and WPL-like motifs that could mediate the interaction with WDR82. This principle is not limited to WDR82 and likely applies to other WD40 repeat proteins. For example, CstF-50, which is part of the mammalian Cleavage Stimulation factor, partly interacts with CstF77 via a motif similar to DPR (DPK) to aid recognition of the G/U-rich elements downstream of PAS and PAS selection by the CPA machinery and cleavage factors^107^. Similarly to WDR82, CstF-50 might mediate links to other complexes and was proposed to interact with BRCA1^108^.

We propose that WDR82 recognises common SLiMs via distinct WDR-interacting motifs that constitute conserved combinatorial WDR82-binding code, which dynamically regulates phosphatase activity, RNA processing, chromatin modification, and transcription termination. Our work establishes WDR82 as a central integrator of transcriptional control and uncovers a general principle by which WD40 repeat proteins organise and couple fundamental cellular processes.

## Methods

### Acronyms and abbreviations

Acronyms and abbreviations used are provided in Supplementary Table 1.

### Strain construction

*S. pombe* strains were grown in YES medium at 30 °C to OD_600_ = ~0.5, unless otherwise stated. DNA cassettes containing the insert with a selectable marker flanked by homology arms enabling targeted integration at endogenous loci were transformed into cells to introduce deletions or tags. To introduce mutations, gene deletions were complemented with wild-type or mutant alleles expressed from ectopic loci (*leu1*). To achieve conditional depletion of Symplekin/Pta1, cells expressing mAID-FLAG-Pta1 were grown in YES with 1 mM auxin (Sigma) at 25 °C. A complementary copy of V5-tagged Pta1 (WT or DPR-AAA mutant) was integrated at the *ura4* locus under a native promoter. Parental strains were used as controls where appropriate. A full list of strains and oligos used for generating strains and plasmids in this study is provided in Supplementary Data 2, and are available from the corresponding author upon request.

### Immunofluorescence

Immunofluorescence of PNUTS-FTP was carried out as published^33,37^. Briefly, 9 mL of cell culture (OD_600_ = 0.5) was fixed by the addition of 1 mL 37% formaldehyde for 30 min at RT. Cells were washed three times with PEM (100 mM piperazine-N,N′-bis[2-ethanesulfonic acid] (PIPES), 1 mM EGTA, 1 mM MgSO_4_, pH 6.9) and treated with 12.5 µL 10 mg/mL zymolyase T100 (9329.2, Carl Roth) in 500 µL PEMS (PEM with 1.2 M sorbitol) for 1 h at 37 °C. Cells were permeabilised in 500 µL PEMS, 1% Triton X100 for 30 s, washed three times with PEM, then incubated on a rotating wheel for 30 min in 200 µL PEMBAL (PEM with 1% bovine serum albumin, 100 mM lysine hydrochloride, 0.1% NaN_3_) at RT. 1/5th of the total fixed cells were used per condition. Cells were incubated with α-FLAG (Sigma, F3165, 1:200) in 100 µL PEMBAL at 4 °C overnight, then washed twice with PEMBAL, followed by incubation in 200 µL PEMBAL for 30 min at RT. Cells were stained with Alexa Fluor 488-conjugated Goat α-Mouse IgG (H + L) Cross-Adsorbed Secondary Antibody (Invitrogen, A11001, 1:400) in 100 µL PEMBAL at 4 °C overnight in the dark, washed consecutively with PEMBAL and PBS, then resuspended in 40 µL PBS. Next, 3 µL of cell suspension were freshly mounted on poly-lysine-coated coverslips, left to dry, and imaged under ROTIMount FluorCare DAPI (HP20.1, Carl Roth). Z-stacks were acquired on a Deltavision Ultra High-Resolution Microscope (Cytiva) using AcquireUltra (1.2.2) and deconvolved with softWoRx (7.2.1) using default settings. Image quantification was performed in Fiji^109^ (ImageJ 1.53t) using a semiautomated ImageJ macro script (FISHquant_v6.ijm; https://github.com/Kilchertlab/Microscopy-analysis). In short, cellular segmentation was performed by thresholding the transmitted-light channel (cell outlines) and the DAPI stain (nuclei). FLAG signal was measured on average intensity Z-projections for nucleus and cytoplasm (total cell without nucleus), and the ratio of mean nuclear fluorescence intensity over mean cytoplasmic fluorescence intensity was calculated for each cell.

### Purification of native PP1–3 × FLAG

A cell pellet of FLAG-tagged PP1 strain grown in 48 L of 2× YES was resuspended in Lysis Buffer (LB) (50 mM Tris pH 7.9, 10% glycerol, 0.5 mM MgCl₂), supplemented with 0.25% Triton X100, 150 mM NaCl, SuperNuclease (SinoBiological), EDTA-free Protease Inhibitor tablets (Roche) and lysed by French Press operated at 35 kPSI. The lysate was then supplemented with 0.1% Triton and 1 mM phenylmethylsulfonyl fluoride (PMSF), followed by centrifugation for 30 min at 34,000 × *g* at 4 °C. The cleared lysate was incubated for 1 h with 2.5 mL of M2 resin (Sigma-Aldrich) at 4 °C. Proteins were eluted using 5 mL 1 mg/mL FLAG peptide in LB. The eluate was diluted with 20 mM NaCl and 1 mM β-mercaptoethanol, and loaded onto a 5 mL HiTrap Q HP column (Cytiva). Elutions were performed with a gradient of 20 mM–1 M LB over 40 CV. Fractions containing PP1–3 × FLAG were pooled, concentrated, snap-frozen in liquid nitrogen and stored at −80 °C.

### Purification of native PNUTS and Symplekin

FTP-tagged PNUTS and V5-tagged Symplekin-expressing fission yeast cells were disrupted in a freezer mill (SPEX SamplePrep) in liquid nitrogen. 5 g of cell powder was resuspended in 25 mL of LB with 100 mM NaCl and 0.5 mM β-mercaptoethanol, 1 mM PMSF, cOmplete Protease Inhibitor Cocktail (Roche) and phosphatase inhibitors: 1 mM NaF, 1 mM sodium orthovanadate, 2 mM imidazole, 1 mM sodium pyrophosphate decahydrate, 0.5 mM glycerol-phosphate, 5 µM cantharidin, 5 nM calyculin A. All following procedures were performed at 4 °C. The lysates were incubated for 30 min and cleared at 40,000 × *g* for 20 min. Following centrifugation, the lysates were incubated with 250 µL of an equilibrated V5-Trap® Magnetic Particles M-270 (ChromoTek) slurry for 1 h. Beads were washed 10 × 1 mL of LB and proteins were eluted twice with 150 µL of 0.2 M glycine pH 2.5. Samples were neutralised with 25 µL of 1 M Tris. Lysate was incubated for 2 h at 4 °C with 1 mL of IgG Sepharose beads (GE Healthcare). The beads were washed four times with 20 mL of LB 100 mM NaCl and elutions performed by incubation with 65 μL of TEV protease (0.5 mg) in 1 mL of LB for 1.5 h at 16 °C. The sample was collected from the beads by centrifugation and incubated with 200 μL of M2 resin anti-FLAG (Sigma) beads for 1 h. Beads were washed (3 × 5 mL of buffer LB 100 mM NaCl), and the elution was performed with 100 μL of glycine buffer. The mixture was neutralised with 20 μL 1 M Tris. The samples were denatured with 8 M urea, reduced with TCEP, alkylated with 2-chloroacetamide, and digested with LysC and trypsin. The reactions were stopped with formic acid and subjected to analysis by mass spectrometry.

### Recombinant protein expression and purification from *E. coli*

#### Recombinant PP1

A codon-optimised *S. pombe* PP1/Dis2 (1–300 aa) coding sequence was cloned into pTXB1 to generate an N-terminal His₆ tag and a C-terminal Mxe GyrA intein-CBD fusion. The plasmid was transformed into *E. coli* BL21 cells co-expressing GroEL/GroES (pGRO7). Cultures were grown in 2 × TY medium supplemented with 30 µg/mL chloramphenicol, 50 µg/mL ampicillin, and 2 mM MnCl₂ at 37 °C. At OD_600_ = 0.5, GroEL/GroES expression was induced with 1 g/L L-arabinose and the temperature was reduced to 10 °C. When cells reached OD_600_ = 1.0, PP1 expression was induced with 0.1 mM IPTG for 48 h at 10 °C. Cells were harvested, resuspended in fresh 2 × TY medium containing MnCl₂ and chloramphenicol, incubated for 2 h at 10 °C, and stored at −70 °C. Pellets were lysed by sonication in LB (L) (25 mM HEPES pH 7.9, 500 mM NaCl, 10% glycerol, 1 mM MnCl₂) supplemented with 5 mM imidazole, 2 mM PMSF and EDTA-free protease inhibitors. Clarified lysates (40,000 × *g*, 20 min, 4 °C) were applied to Talon Superflow resin (Takara Bio). Resin was washed and protein eluted with buffer L with 250 mM imidazole, after which elution was tenfold diluted in buffer L with 50 mM β-mercaptoethanol and Intein self-cleavage was performed at 22 °C for 72 h. To remove uncleaved material the sample was re-applied to Talon resin and elution was dialyzed overnight at 4 °C into buffer L with 1 mM β-mercaptoethanol, snap-frozen in 40% glycerol, and stored at −70 °C.

#### Recombinant PNUTS

A gene fragment corresponding to PNUTS residues 420 to 710 containing an N-terminal His₆ tag and a C-terminal Twin-Strep tag was cloned into a modified pTXB1 vector lacking intein-CBD. Mutant ${{{\rm{PNUTS}}}}_{420-710}^{{{\rm{W}}}510{{\rm{A}}}}$ was generated by PCR mutagenesis. Proteins were expressed in *E. coli* Rosetta (DE3) cells grown in 2 × TY medium supplemented with chloramphenicol (25 µg/mL) and ampicillin (50 µg/mL) at 37 °C. 0.3 mM IPTG was added to cells at an OD_600_ = 1.0, and they were incubated at 20 °C overnight. Cell pellets were lysed in Strep buffer (50 mM HEPES pH 7.9, 150 mM NaCl, 0.5 mM MgCl₂, 1 mM β-mercaptoethanol) supplemented with 2 mM PMSF and protease inhibitors. Lysates were clarified by centrifugation (40,000 × *g*, 20 min, 4 °C) and filtration (0.22 µm) and applied to a 5 mL Strep-Trap HP column (Cytiva). Bound protein was eluted with Strep buffer containing 5 mM desthiobiotin (IBA Lifesciences) and subsequently incubated with Talon Superflow resin. After incubation, the resin was washed sequentially with Strep buffer containing 5 mM Mg²⁺-ATP and with 2 mM imidazole. Proteins were eluted using 250 mM imidazole. Final purification was performed by size-exclusion chromatography (HiLoad 16/600 Superdex 75, Cytiva) equilibrated in gel-filtration buffer (25 mM HEPES, pH 7.9, 500 mM NaCl, 10% glycerol, 1 mM MgCl₂, 1 mM β-mercaptoethanol). Peak fractions were concentrated using 10 kDa MWCO centrifugal filters, snap-frozen, and stored at −70 °C.

#### Spt5-Kow_5_-CTR_7_

N-terminally His-tagged Spt5-Kow_5_-CTR_7_ was expressed in *E. coli* as previously described ^37^. Briefly, cleared lysate was affinity purified over Ni-NTA resin (Qiagen), followed by Heparin Sepharose (HiTrap Heparin HP, Cytiva) and HiLoad 16/600 Superdex 75 column size exclusion chromatography. The Spt5-Kow5-CTR_7_ (54 μM) was phosphorylated with Cdk9_1–350_-Cyclin T (Pch1) kinase complex (0.4 μM) in 1 mM MgCl₂, ATP and MnCl₂ in a total volume of 1 mL Strep buffer. The reaction was incubated for 1.5 h at 30 °C and then loaded onto a HiLoad 16/600 Superdex 75. Peak fractions were combined and concentrated using an Amicon 10 kDa Ultra Centrifugal Filter (Millipore) and stored at −70 °C. The phosphorylation status of the Spt5 was evaluated by 10% Phos-tag resolving gel (2.5 µM Phos-tag Acrylamide and 50 µM MnCl₂). 

### Recombinant protein expression from *Sf9* cells

All proteins expressed from Sf9 were cloned into pACEBac1. Bacmids were generated by transformation of DH10EmBacY cells and positive clones were screened by blue-white screening. Bacmids were extracted from 3 mL overnight cultures using isopropanol precipitation. 2 mL of Sf9 cells at 0.5 × 10^6^ were transfected with 2 μg of bacmid using FuGene transfection reagent (Promega). After 5 days at 27 °C, cells were harvested and the supernatant was filtered using a 0.22 μm filter. P2 virus was generated by infection of Sf9 cells at 1.5–2.0 × 10^6^ with P1 virus at a 1:100 ratio (v/v). After 4 days at 27 °C, cells were harvested and supernatant was filtered using a 0.22 μm filter and stored at 4 °C in the dark. Large-scale protein expression was carried out by infecting Sf9 cells at 2.0 × 10^6^ with P2 virus at a 1:100 ratio (v/v). Cells were harvested after 3 days at 27 °C, washed with PBS, and stored at −80 °C.

PNUTS, PNUTS^WPL-G/A^ and PNUTS-WDR82 were purified with the following protocol. Cells were resuspended in Strep Buffer supplemented with SuperNuclease, Protease Inhibitors (Roche), and 2 mM PMSF, and lysed by sonication. The lysate was clarified by centrifugation using a JA25.5 rotor at 40,000 × *g* for 20 min at 4 °C. The clarified lysate was filtered through a 0.22 μm filter and loaded onto a 5 mL StrepTrap HP column equilibrated with Strep Buffer. The column was washed with 20 CV of Strep buffer before elution with Strep Elution Buffer with 5 mM Desthiobiotin (IBA Sciences). The eluate was diluted to 30 mM NaCl and loaded onto a 5 mL HiTrap Q HP equilibrated with QA buffer (50 mM HEPES pH 7.9, 30 mM NaCl, 0.5 mM MgCl₂, 1 mM β-mercaptoethanol). Protein was eluted with a gradient of 0–100% QB buffer (50 mM HEPES pH 7.9, 1 M NaCl, 0.5 mM MgCl₂, 1 mM β-mercaptoethanol) over 40 CV. Fractions were pooled, concentrated, and applied onto a Superdex 200 Increase 10/300 GL column (Cytiva). Peak fractions were pooled, concentrated, and snap-frozen in liquid nitrogen.

WDR82, PNUTS_WBR_-WDR82, Symplekin_NTD_-Ssu72, and ${{{\rm{Symplekin}}}}_{{{\rm{NTD}}}}^{{{\rm{DPR}}}-{{\rm{AAA}}}}$-Ssu72 were purified essentially as described above except for the following modifications. Cells were resuspended in Strep Buffer supplemented with 2 mM PMSF, 2.4 mL/L cell culture of Biolock (IBA), and protease inhibitors. Cells were lysed in the presence of 0.5 % Triton-X100. Lysate was clarified by centrifugation and applied to 3 mL Streptactin XT 4 Flow High-Capacity resin (IBA). Resin was washed with 10 CVs of Strep Buffer 3 times followed by elution with 50 mM Biotin (IBA) in Strep Buffer. The C-terminal StrepII-tag was cleaved by addition of home-made TEV protease at a 1:20 target protein to TEV protease ratio and incubation at 4 ^°^C overnight. Complexes were then purified by anion exchange and size exclusion chromatographies as described above. Peak fractions were pooled, concentrated, and either used immediately for crystallisation trials or flash frozen in liquid nitrogen.

To reconstitute the PNUTS_WBR_-WDR82-Symplekin_NTD_-Ssu72 complex for crystallisation, PNUTS_WBR_-WDR82 and Symplekin_NTD_-Ssu72 dimers were expressed and purified separately by Streptactin affinity and anion exchange chromatography as described above. Subcomplexes purified from anion exchange chromatography were concentrated and mixed with PNUTS_WBR_-WDR82 at a slight molar excess (molar ratio of 1:1.2) for 15 min on ice in Strep Buffer, followed by purification on a Superdex 200 column. Peak fractions were pooled, concentrated to ~ 17 mg/mL, and used for crystallisation trials.

To prepare the 10-subunit CPF, Sf9 cells were co-infected with the Polymerase Module (CPSF160/Cft1, CPSF30/Yth1, FIP1L1/Iss1, WDR33/Pfs2-Strep tag and PAPOLA/Pla1) and the Nuclease and Phosphatase Modules without PNUTS, WDR82, and PP1 (Ysh1, Cft2, Pta1-Strep, Ctf1, Ssu72) baculoviruses at a 1:1 ratio (v:v) relative to each other (total virus:culture ratio of 1:50 (v:v)). Cells were harvested 72 h post-infection and resuspended in Strep Buffer. CPF was purified by Streptactin and anion exchange chromatographies as described above except that home-made TEV protease was added to the Streptactin affinity eluate at a 1:20 ratio (w:w) and the twin-StrepII tags on Symplekin and WDR33 were cleaved overnight at 4 °C. During anion exchange chromatography, the polyA polymerase Pla1 (PAPOLA in human) dissociates and elutes as a distinct peak at lower salt. Fractions corresponding to PAPOLA and the rest of core CPF were pooled and the salt concentration diluted back down to 150 mM. The complex was then concentrated to <100 µL and injected onto a Superose 6 Increase 3.2/300 GL column (Cytiva) equilibrated in Strep Buffer. Peak fractions were pooled, concentrated, and flash-frozen in liquid nitrogen.

### Crystallisation and structure determination

#### PNUTS_WBR_-WDR82

PNUTS_WBR_-WDR82 at 9.85 mg/mL was crystallised using the sitting-drop vapour diffusion set-up at 20 °C. Crystals used for structure solution appeared overnight in two conditions: the first condition was 0.05 M Calcium chloride dihydrate, 0.1 M MES monohydrate pH 6.0, 45% v/v Polyethylene glycol 200, while the second condition was 10% w/v PEG 8000, 20% v/v ethylene glycol, 0.03 M of each MES/imidazole pH 6.5. Crystals reached full size after a week and were harvested without addition of cryoprotectant before flash freezing in liquid nitrogen. X-ray diffraction data were collected on the I04 beamline at Diamond Light Source (Harwell, UK). Data collection, refinement statistics and a representative portion of the electron density map for each structure are provided in Supplementary Data 3. Data were automatically processed with autoPROC/STARANISO^110^. The structure was solved via molecular replacement in PHASER^111^, using an AlphaFold2 generated model of WDR82 as the initial search model^112^. PNUTS_WBR_ was built manually into the electron density in COOT^113^.

#### PNUTS_WBR_-WDR82-Symplekin_NTD-_Ssu72

PNUTS_WBR (565-644)_-WDR82-Symplekin_1–307-_Ssu72 at 17 mg/mL was crystallised using the sitting-drop vapour diffusion set-up at 20 °C. Small crystals appeared in a condition containing 100 mM MOPS pH 7.5, 100 mM magnesium acetate, and 12% PEG 8000 after 9 days. The drop was composed of 200 nL protein and 100 nL reservoir solution. The crystals were resuspended in 50 μL of the reservoir solution and crushed by vortexing 3 times for 30 s using a plastic bead. The seed stock was diluted 1:2 in reservoir solution and used for microseed sparse-matrix screening in sitting-drop vapour diffusion set-up at 20 °C. Crystals appeared after 2–4 days in multiple conditions and grew to maximal size after 10–14 days. Crystals were cryoprotected with 30% glycerol before flash freezing in liquid nitrogen. X-ray diffraction data were collected on the I04 beamline at Diamond Light Source (Harwell, UK) (Supplementary Data 3). Data were automatically processed with autoPROC/STARANISO. The structure was solved via molecular replacement in PHASER. The input models were the experimentally solved crystal structure of PNUTS_WBR_-WDR82 (PDB: 9SCE) and AlphaFold2-generated models of Symplekin_NTD_ and Ssu72. The model was iteratively refined in PHENIX^114^, followed by manual rebuilding in COOT^113^.

### Analytical size exclusion chromatography (SEC)

Small-scale reconstitution experiments were performed in total reaction volumes of 50 μL in Strep Buffer. Individually purified WDR82, Symplekin_NTD_-Ssu72, ${{{\rm{Symplekin}}}}_{{{\rm{NTD}}}}^{{{\rm{DPR}}}-{{\rm{AAA}}}}$-Ssu72, PNUTS_WBR_-WDR82, as described above, were mixed in the combinations described for final protein concentrations of 64.2 μM in 50 μL total reaction volumes, incubated for 15 min on ice and loaded onto a Superdex 200.

### XL-MS of PNUTS-WDR82-Symplekin-Ssu72

PPW-Symplekin-Ssu72 isolated by size exclusion chromatography (10 μg), was crosslinked with 8 mM BS3 on ice for 1 h. Samples were then quenched with 100 mM Tris, pH 7.5. Protein was denatured by incubating with 4 M urea for 10 min at room temperature (RT), 675 rpm shaking. Samples were then incubated with 10 mM TCEP (Tris(2-carboxyethyl)phosphine) for 30 min at RT to reduce cysteines, incubated with 50 mM chloroacetamide (CIAM) for 30 min in the dark at RT, and pre-digested using LysC (1 μg LysC per 100 μg sample) for 2 h at 37 °C, shaking at 800 rpm. 100 mM Ammonium Bicarbonate was then added to the sample to dilute urea to 2 M and CaCl_2_ to 2 mM, and the sample was then digested with trypsin at a ratio of 1 μg per 40 μg sample, incubated for 16-20 h at 37 °C, shaking at 800 rpm. Trypsin digestion was quenched with 5% formic acid and stored at −20 °C.

### LC-MS/MS analysis

Dried peptides were resuspended into 5% acetonitrile/5% formic acid before LC-MS/MS analysis. Peptides were separated by nano liquid chromatography (Ultimate RSLC 3000) coupled in line to a QExactive mass spectrometer equipped with an Easy-Spray source (Thermo Fisher Scientific). Peptides were trapped onto a C18 PepMac100 precolumn (300 µm i.d. × 5 mm, 100 Å, ThermoFisher Scientific) using Solvent A (0.1% Formic acid, HPLC grade water). The peptides were further separated onto an Easy-Spray RSLC C18 column (75 µm i.d., 50 cm length, Thermo Fisher Scientific) using a 30 (Symplekin^WT^ and Symplekin^DPR-AAA^ mutant), 60 (PNUTS-FTP-TANDEM), 120 (PPW-Symplekin-Crosslinking) and 180 min (PPW-STABLE-COMPLEX) linear gradient (15–35% solvent B (0.1% formic acid in acetonitrile)) at a flow rate of 200 nl/min. The raw data were acquired using the mass spectrometer in data-dependent acquisition mode. Full-scan MS spectra were acquired in the Orbitrap (Scan range 350–1500 m/z, resolution 70,000; AGC target, 3e6, maximum injection time, 50 ms). For 30/60 min and 120/180 min gradients, the 10 and 20 most intense peaks were selected for higher-energy collision dissociation (HCD) fragmentation at 30% of the normalised collision energy. HCD spectra were acquired in the Orbitrap at resolution 17,500, AGC target 5e4, maximum injection time of 120 ms with fixed mass at 180 m/z. Charge exclusion was selected for unassigned and 1+ ions. The dynamic exclusion was set to 5, 20, 40 and 60 s for 30-, 60-, 120- and 180-min gradients, respectively.

### Mass spectrometry data processing

For protein identification for PNUTS/Ppn1-FTP, tandem mass (MS/MS) spectra were searched using Sequest HT in Proteome Discoverer software version 1.4 against a protein sequence database containing 5507 protein entries, including 5224 *S. pombe* proteins (UniProt release from 21-11-23), using FLAG-tagged PNUTS protein sequence and 283 common contaminants. During database searching, cysteines (C) were fully carbamidomethylated (+57.0215, statically added), methionine (M) was fully oxidised (+15.9949, dynamically added), and all N-terminal residues were acetylated (+42.0106, dynamically added). Two missed cleavages were permitted. Peptide mass tolerance was set at 50 ppm on the precursor and 0.6 Da on the fragment ions. Data were filtered at an FDR below 1% at the PSM level. For cross-linked peptides identification, tandem mass spectra were searched using pLink software^115^ version 2.3.9 against a protein sequence database containing *Spodoptera frugiperda* protein sequences (UniProt release from 21-02-26) combined with user-defined tagged protein sequences of interest, and 283 common contaminants. During pLink searches, BS3 crosslinker was selected, cysteines (C) were considered to be fully carbamidomethylated (+57.0215, statically added), methionine (M) to be fully oxidised (+15.9949, dynamically added), all N-terminal residues to be acetylated (+42.0106, dynamically added). Three missed cleavages were permitted. Peptide mass tolerance was set at 20 ppm on the precursor and fragment ions. Data were filtered at False Discovery Rate (FDR) below 5% at PSM level and summarised in Supplementary Data 4.

### In vitro dephosphorylation assay

PP1 activity was tested using either PP1 alone (4 μM), or PP1 (4 μM) mixed with PNUTS_420–710_ (8 μM) with or without WDR82 (8 μM), and was pre-incubated at 25 °C for 15 min. Next, samples were diluted in Reaction Buffer (20 mM HEPES pH 7.5, 100 mM NaCl, 2 mM MnCl₂, 1 mM β-mercaptoethanol) to final concentrations of 20 nM PP1 and 40 nM PNUTS_420–710_ or WDR82. Dephosphorylation reactions were performed at 25 °C and initiated by addition of CTR_1_, CTR_4_ or Spt5-Kow_5_-CTR_7_ (Kow5 with seven CTR repeats (Spt5-Kow_5_-CTR_7_) in vitro phosphorylated by recombinantly reconstituted *S. pombe* Cdk9/Cyclin T1)^33,37^ or short phospho-CTR-peptides (1 and 4 repeats, Peptide Protein Research Ltd) substrates at final concentrations of 80, 20 and 12 μM, respectively and quenched with Malachite Green working reagent (Sigma-Aldrich) at specific time points. Phosphate release was quantified by measuring absorbance at 620 nm on a PHERAstar FS plate reader (BMG Labtech) or a DeNovix spectrophotometer and converting values to concentrations using a standard curve generated from known amounts of inorganic phosphate. Negative values were offset to zero. Measurements deviating by more than 100% from the mean of replicate samples at the same time point were classified as outliers and excluded. Curves were fitted using non-linear least squares regression in R (v4.3.3) with the nls function; for reactions using the Spt5-Kow_5_-CTR_7_ substrate, where nls convergence was not achieved, a LOESS regression (loess function) was applied. Statistical differences among groups at the 60-min time point were assessed using the Kruskal–Wallis rank-sum test, followed by Dunn’s post hoc test with Bonferroni correction when appropriate. All analyses were performed in *R* (version 4.3.3) environment.

### Pulldown assays

For the pulldown assay to test for interaction between the core CPA and PPW complex, all proteins or protein complexes were recombinantly expressed and purified from Sf9 cells, except for PP1, which was purified from fission yeast cells. Co-expression of 10 CPF subunits in Sf9 cells was performed using two MultiBac constructs.

The assays were performed in Strep Buffer. 0.5 μM twin-Strep II tagged PNUTS or PNUTS-WDR82 complex was mixed with 1 μM CPF, PP1–3 × FLAG, and/or WDR82 in 50 μL total volume. 20 μL Streptactin XT high-capacity resin (IBA Life Sciences) equilibrated in Strep Buffer was used per pulldown and the mixture was incubated at 4 °C for 1 h. Resin was washed twice with 200 μL Strep Buffer supplemented with 0.05% Triton X100. Elutions were analysed by NuPAGE 4–12% gels (Thermofisher).

### TT*-*seq and bioinformatic analyses

TT-seq was performed in duplicates (except for the complementation Symplekin^WT^ strain) following published procedures with minor adjustments^37^. Reads were processed with fastp 0.20.0^116^ and aligned to a combined *S. pombe*/*S. cerevisiae* reference using STAR 2.7.3a^117^. Species-specific reads were separated by chromosome identifiers, and uniquely mapped *S. cerevisiae* reads were obtained with SAMtools 1.19.2^118^ for spike-in normalisation. Metagene analyses and heatmaps were generated using deepTools 3.5.5^119^ (log_2_ ratios, gene body (GB) scaling when indicated), restricting analyses to ~3300 coding genes lacking same-strand neighbours within 250 bp of the TSS (Transcription Start Site) or PAS. Changes in 3′UTR usage were normalised to spike-in BigWig files and quantified as the ratio of UTR to GB signal density for ~4489 pc genes with 3′UTRs ≥ 50 bp. Genes with ≥1.5-fold increase in ≥2 mutants (514 genes) or Symplekin^DPR-AAA^ vs WT complementation strains (526 genes) were classified as upregulated. Hexamer enrichment in upregulated 3′UTRs was assessed by a hypergeometric test with Benjamini–Hochberg FDR correction (significant: FDR < 0.05, |log₂FC| > 1). Transcription termination defects were quantified as increased signal in a 100 bp post-PAS window relative to GB density in 3663 pc genes with 3′UTRs ≥ 50 bp, expression ≥10, and without a gene on the same strand within 250 bp between mutant and WT. Overlap between multi-mutant phosphatases (defects in ≥2 mutants) and Symplekin mutant/WT was evaluated using Venn diagrams and heatmaps showing pairwise percentage overlaps of the smallest set.

## Supplementary information

Supplementary Information

Description of Additional Supplementary Files

Supplementary Data 1

Supplementary Data 2

Supplementary Data 3

Supplementary Data 4

Transparent Peer Review file

## Source data

Source Data

## Data Availability

The model and map for the structures presented in this study have been deposited in the PDB database with the identifiers 9SCE (PNUTS_WBR_—WDR82, crystal 1) and 9SYQ (Symplekin_NTD_-Ssu72-PNUTS_WBR_-WDR82 complex-crystal 1) (PDB https://doi.org/10.2210/pdb9SCE/pdb and PDB https://doi.org/10.2210/pdb9SYQ/pdb). TT-seq data have been deposited in NCBI’s Gene Expression Omnibus and are accessible through GEO Series accession number GSE308656. Standard bioinformatic packages were used throughout the manuscript. Proteomics data were deposited into the PRIDE repository under the dataset identifiers PXD081886 and PXD081924. Source data are provided with this paper.
