# Supplementary material for: WDR82 code coordinates transcription and pre-mRNA 3′-end processing through regulated phosphorylation
Source: Nat Commun. 2026 Sep 15;17:11131. doi: 10.1038/s41467-026-77611-z (PMC13710762; doi:10.1038/s41467-026-77611-z)
Supplement: Supplementary file 1 [file 41467_2026_77611_MOESM1_ESM.pdf]

Supplementary Table 1

Abbreviation	Definition
AAUAAA	Polyadenylation signal hexamer
AID	Auxin-inducible degron
APA	Alternative polyadenylation
APT	Associated with Pta1 complex
BARD1	BRCA1-associated RING domain protein 1
BRCA1	Breast cancer type 1 susceptibility protein
BS3	Bis(sulfosuccinimidyl) suberate
CFIA	Cleavage factor IA
CFIB	Cleavage factor IB
CDK9	Cyclin-dependent kinase 9
CPA	Cleavage and polyadenylation machinery
CPF	Cleavage and polyadenylation factor
CPSF	Cleavage and polyadenylation specificity factor
CTD	Carboxy-terminal domain
CTR	C-terminal region
CstF	Cleavage stimulation factor
DPR	Asp-Pro-Arg motif
DRB	5,6-dichlorobenzimidazole riboside
DSIF	DRB sensitivity-inducing factor
FLAG	Epitope tag (DYKDDDDK)
FTP	FLAG-TEV-Protein A tag
GC	Guanine-cytosine
HUSH	Human silencing hub complex
Integrator	Integrator complex
KOW	Kyrpides-Ouzounis-Woese domain
LMWPTP	Low molecular weight protein tyrosine phosphatase
mCF	Mammalian cleavage factor
nanoESI-MS	Nano-electrospray ionisation mass spectrometry
NELF	Negative elongation factor
NNS	Nrd1-Nab3-Sen1 complex
NTD	N-terminal domain
OD600	Optical density at 600 nm
PAF	Polymerase-associated factor
PAS	Polyadenylation signal
Pol II	RNA polymerase II
PP1	Protein phosphatase 1
PPW	PP1-PNUTS-WDR82 complex
pre-mRNA	Precursor messenger RNA
P-TEFb	Positive transcription elongation factor b
PNUTS	PP1 nuclear targeting subunit
RVxF	Arginine-valine-any-phenylalanine motif
SDS-PAGE	Sodium dodecyl sulfate-polyacrylamide gel electrophoresis
SEC	Size-exclusion chromatography
SET1	Histone H3K4 methyltransferase complex
SLiM	Short linear motif
SU(S)	Suppressor of sable
TT-seq	Transient transcriptome sequencing
UTR	Untranslated region
V5	Epitope tag
WBR	WDR82-binding region
WDR82	WD repeat domain 82
WT	Wild type
XL-MS	Cross-linking mass spectrometry
Xrn2	5′-3′ exoribonuclease 2
ZC3H4	Zinc finger CCCH-type containing 4

Supplementary Figure 1

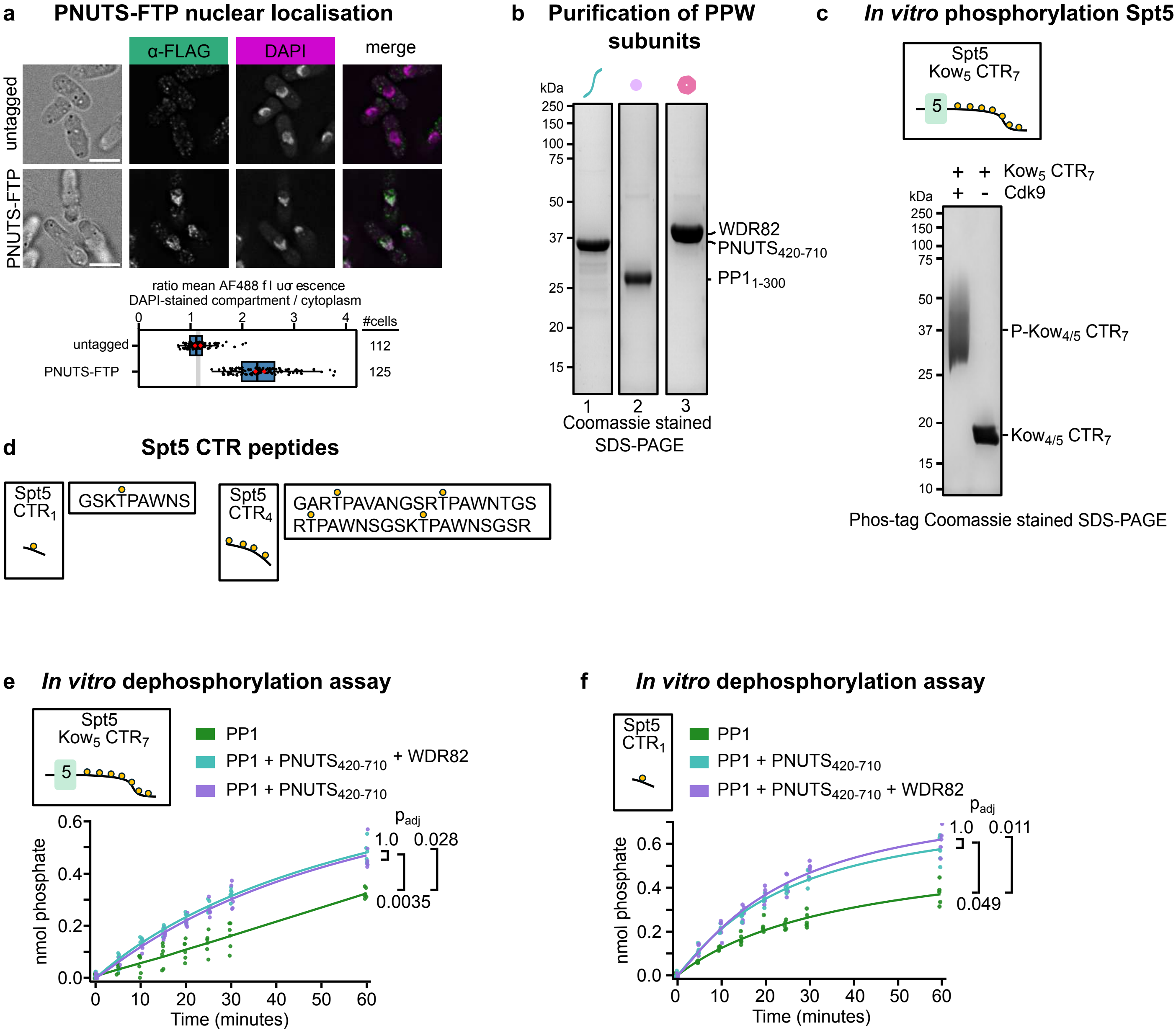

Supplementary Figure 1. PNUTS cellular localisation and purification of the PP1–PNUTS–WDR82 (PPW) phosphatase module components, and their impact on Spt5 CTR dephosphorylation. a Nuclear localisation of *S. pombe* PNUTS. Immunofluorescence against FLAG in untagged WT and PNUTS-FTP cells. The merged channel shows FLAG in green and DAPI in magenta. Scale bar = 5 µm. Bottom: Quantitation of FLAG signal (n= 2). Black horizontal lines mark the median and the thick grey line marks the mean FLAG signal ratio of the untagged strain. **b** Purification of PPW subunits for the phosphatase assay. $PNUTS_{420\text{-}710}$ was purified from *E. coli* by sequential affinity steps and SEC (lane 1). PP1 was purified from *E. coli* using an intein-based system (lane 2). WDR82-STREP was purified from Sf9 insect cells using Strep-Trap HP chromatography (lane 3). **c** Phos-tag gels of Kow_5-CTR_7 phosphorylated by incubation with Cdk9-Pch1. **d** Schematic showing phosphorylated peptide substrate sequences. **e** Phosphatase assay using Kow_5-CTR_7 as a substrate (n=6). Statistical significance was assessed by Kruskal–Wallis test at the 60 min time point, followed by Dunn's post hoc test with Bonferroni correction. Adjusted p-values (p_{adj}) for all three pairwise comparisons are shown. **f** Phosphatase assay using 1× phosphorylated peptide substrate (n=5, Spt5-CTR_1). Statistical analysis was performed as described in panel **e**.

Supplementary Figure 2

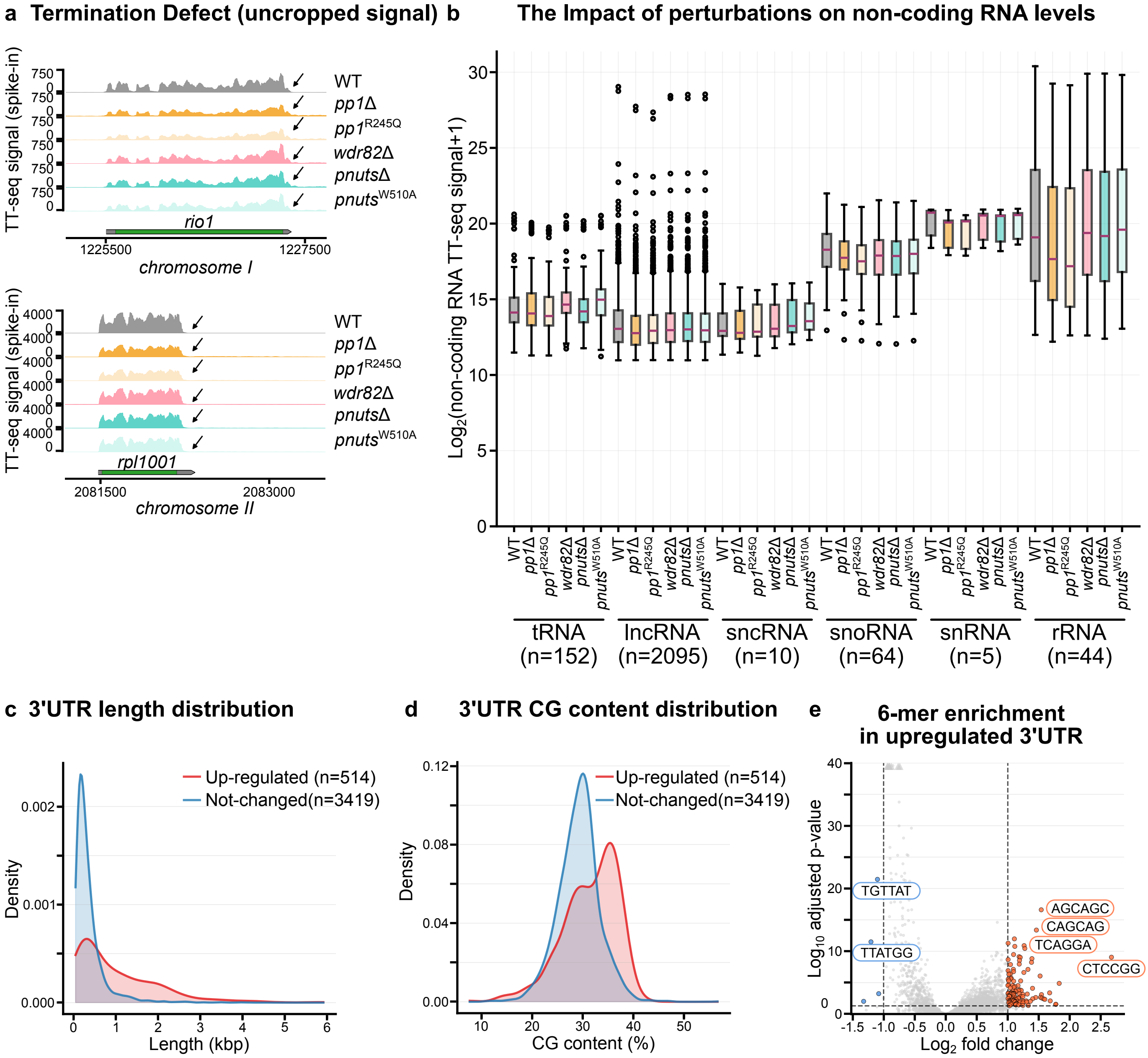

Supplementary Figure 2. Transcriptional changes upon loss of the PP1 phosphatase module subunits.
a Transcriptional readthrough upon loss of PP1, its catalytic activity or regulatory subunits (WDR82, PNUTS or complementation strain with PNUTS^{W510A} which loses interaction with PP1). Uncropped TT-seq signal in indicated mutants, related to Figure 2b (n=2). **b** Changes in different classes of non-coding RNAs in mutants are described in **a**. Boxes span the interquartile range (25th-75th percentile), the centre line is the median, and whiskers extend to the most extreme value within 1.5 × IQR of the box edges. Circles are outliers beyond the whiskers. **c** Distribution of 3'UTR lengths, which were upregulated at least 1.5-fold in at least 2 mutants described in **a**, n=514, compared to 3'UTRs that have not been assigned to be changed in any of the mutants (n=3419). **d** Comparison of 3'UTRs that were upregulated (as in **c**) to the control. **e** Hexamer enrichment analysis for sequences found in upregulated 3'UTRs versus the unchanged set (as in **c**–**d**).

Supplementary Figure 3

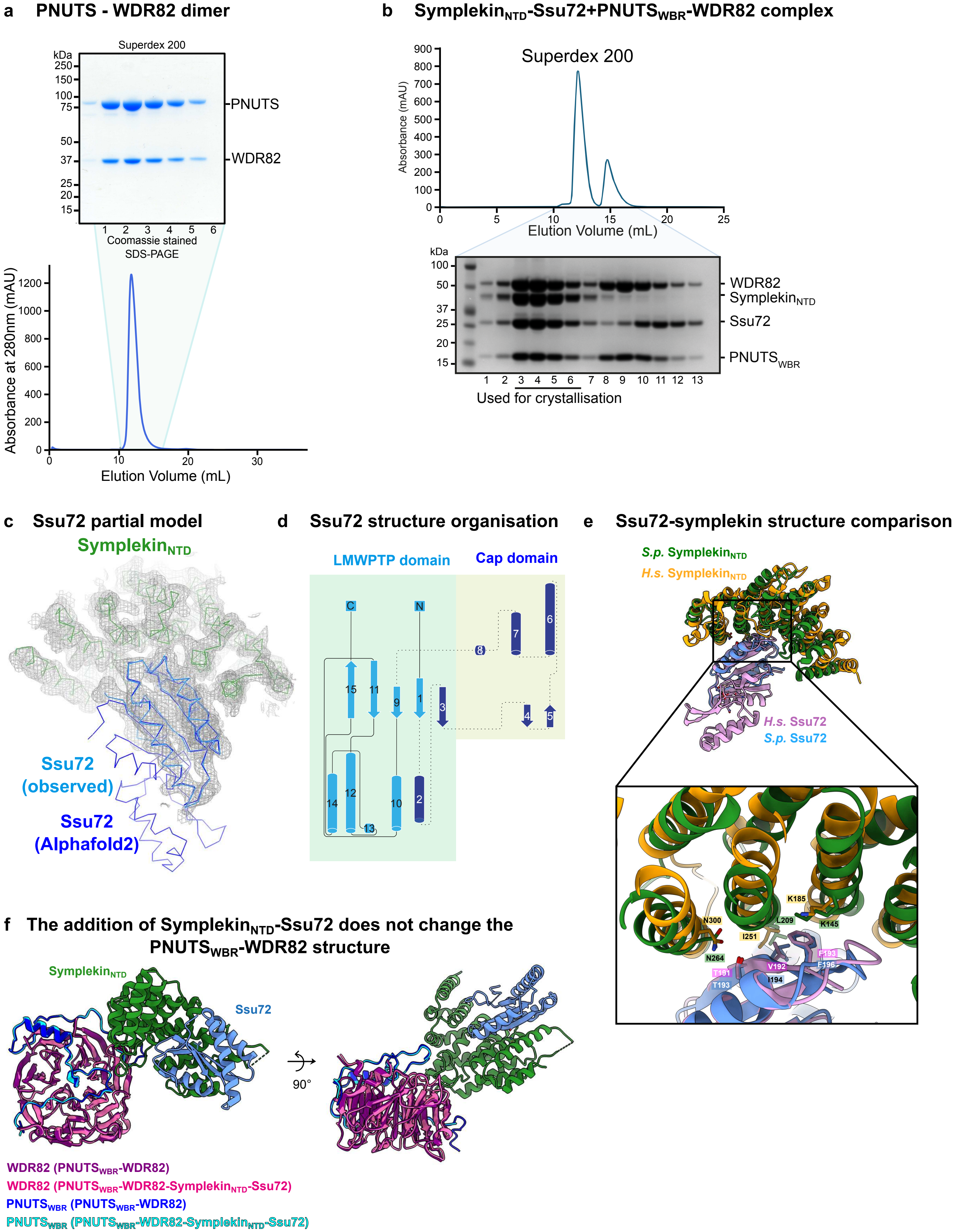

Supplementary Figure 3. Reconstitution and structural characterisation of the $PNUTS_{WBR}$–WDR82–$Symplekin_{NTD}$–Ssu72 complex. **a** PNUTS and WDR82 were co-expressed in insect cells and analysed by size-exclusion chromatography, revealing co-elution as a stable heterodimeric complex. **b** Purified $PNUTS_{WBR}$-WDR82 and $Symplekin_{NTD}$-Ssu72 heterodimers were combined and resolved by size-exclusion chromatography, yielding a stable reconstituted hetero-tetrameric complex. **c** Electron density encapsulating Ssu72 and $Symplekin_{NTD}$ in the tetramer structure. The only part of Ssu72 that is present in the crystal structure corresponds to the LMWPTP domain. The partial Ssu72 model is overlaid on the AlphaFold-predicted structure. **d** A Ssu72 protein topology diagram highlighting CAP and LMWPTP domains. The tetrameric structure only contained density covering part of the LMWPTP domain. **e** Ssu72 interaction with Symplekin is conserved. Structural superimposition of $PNUTS_{WBR}$-WDR82-$Symplekin_{NTD}$-Ssu72 (this work) with Symplekin-Ssu72 (human, PDB ID: 3O2S). **f** The $PNUTS_{WBR}$-WDR82 heterodimer maintains the same overall conformation in $PNUTS_{WBR}$-WDR82-$Symplekin_{NTD}$-Ssu72 tetramer as observed when the $PNUTS_{WBR}$-WDR82 structure was solved independently.

Supplementary Figure 4

a Density around the DPR motif

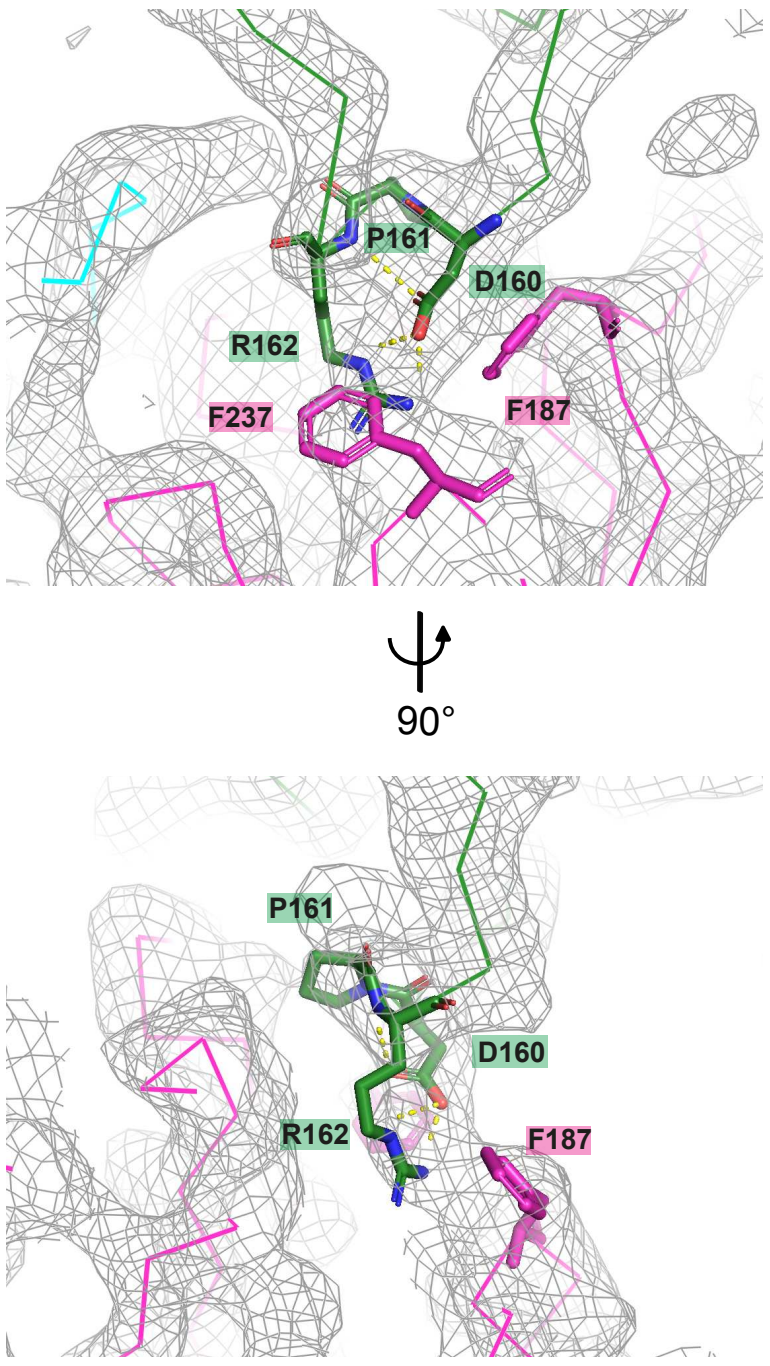

b Size Exclusion chromatography

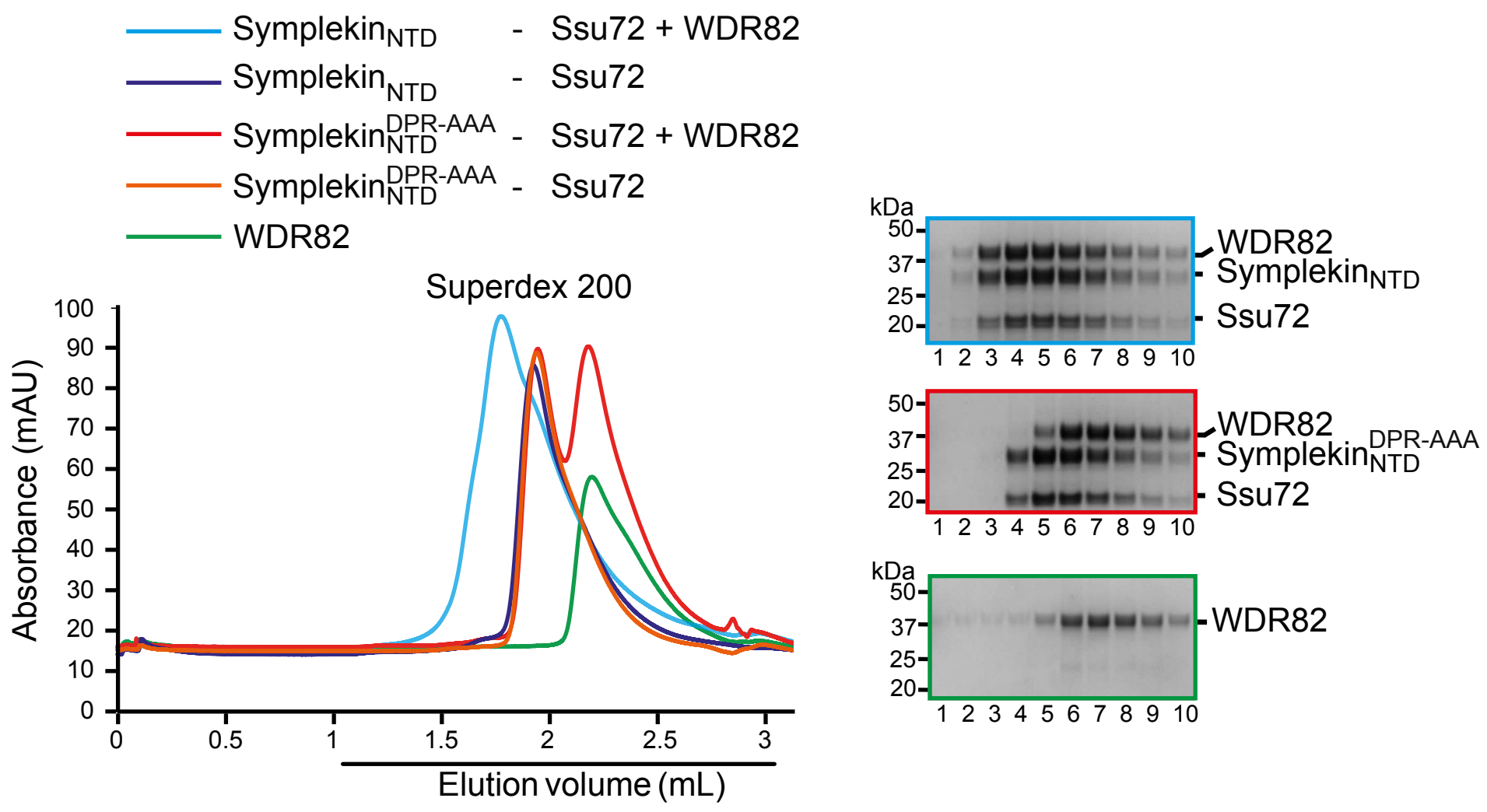

c PNUTS constructs used in pulldown assay

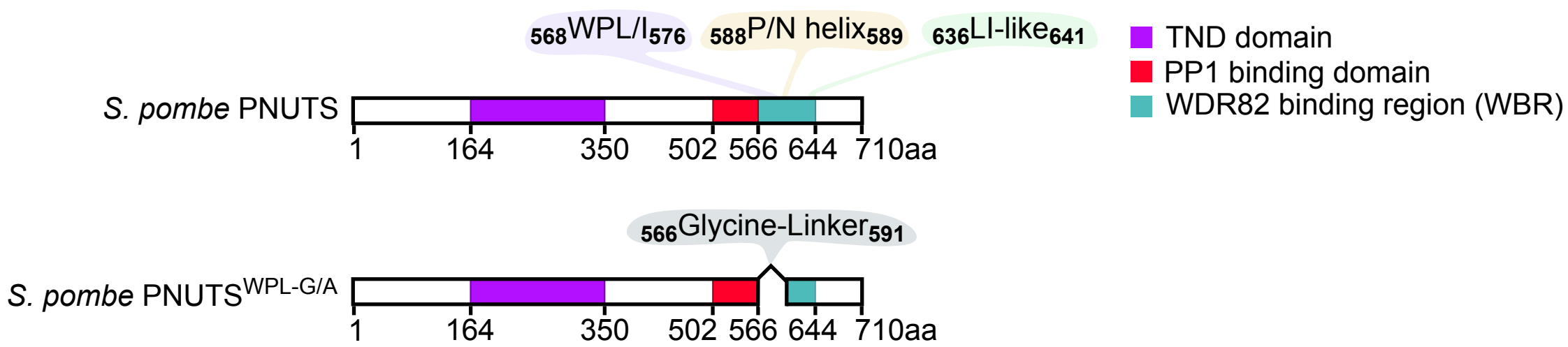

d Probing the PPW interaction with Symplekin-Ssu72

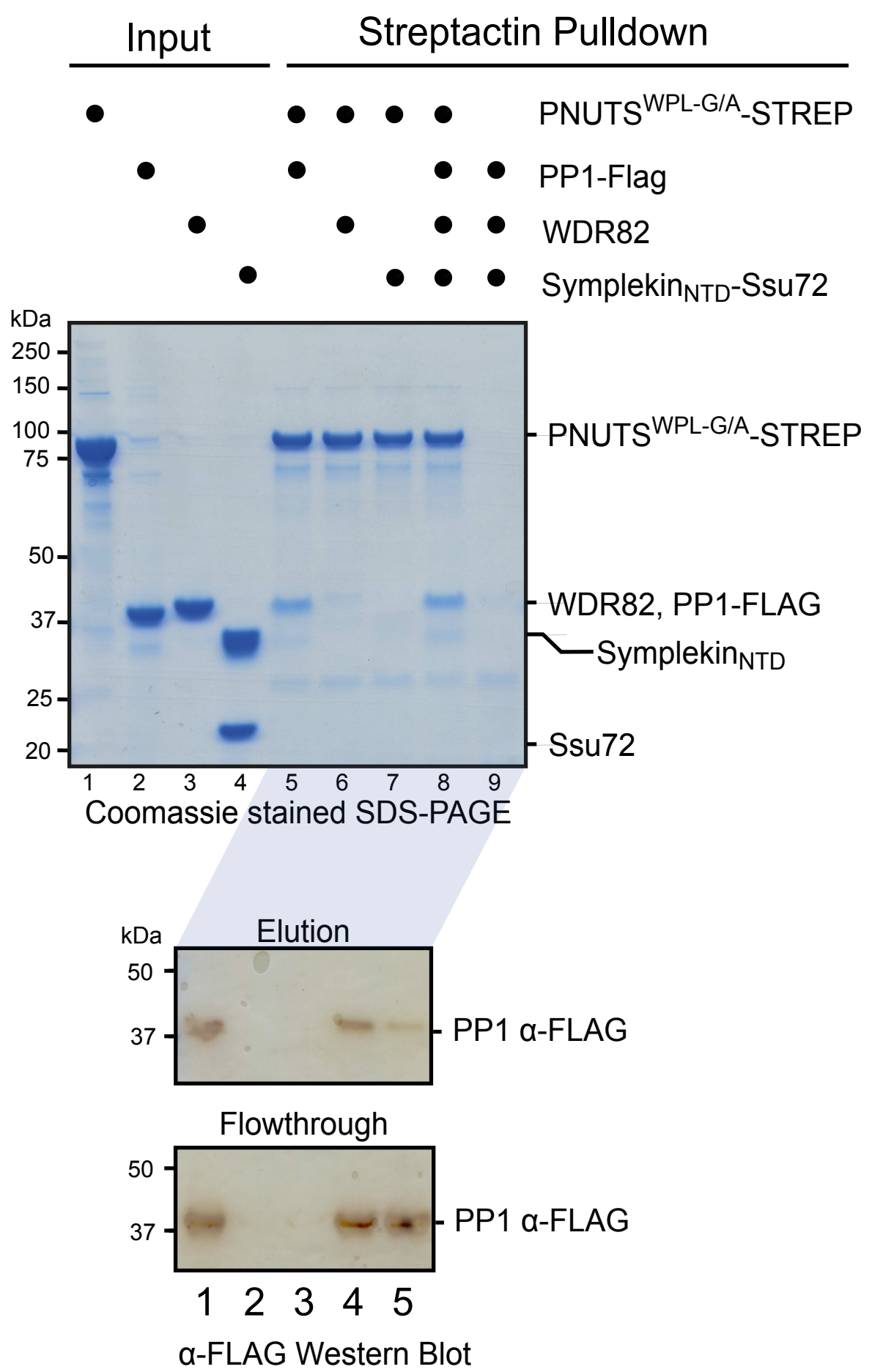

Supplementary Figure 4. The Symplekin DPR and PNUTS WPL motifs are required for assembly of the phosphatase module. a Electron density map covering the interface between the Symplekin DPR motif and the WDR82 binding pocket. **b** Analyses of the complex for WDR82 with Symplekin$_{NTD}$-Ssu72 or Symplekin$_{NTD}^{DPR-AAA}$-Ssu72 formation by analytical SEC (Superdex 200). The Symplekin$^{DPR-AAA}$ mutant formed a heterodimer with Ssu72; however, it failed to assemble with WDR82 into the heterotrimer, in contrast to wild-type Symplekin$_{NTD}$-Ssu72.
c Domain maps *S. pombe* PNUTS and PNUTS$^{WPL-G/A}$. **d** PNUTS$^{WPL-G/A}$ mutant retained PP1 binding but failed to pull down Symplekin$_{NTD}$-Ssu72, even with WDR82 present, highlighting the importance of the WDR82-interacting surface in complex assembly.

Supplementary Figure 5

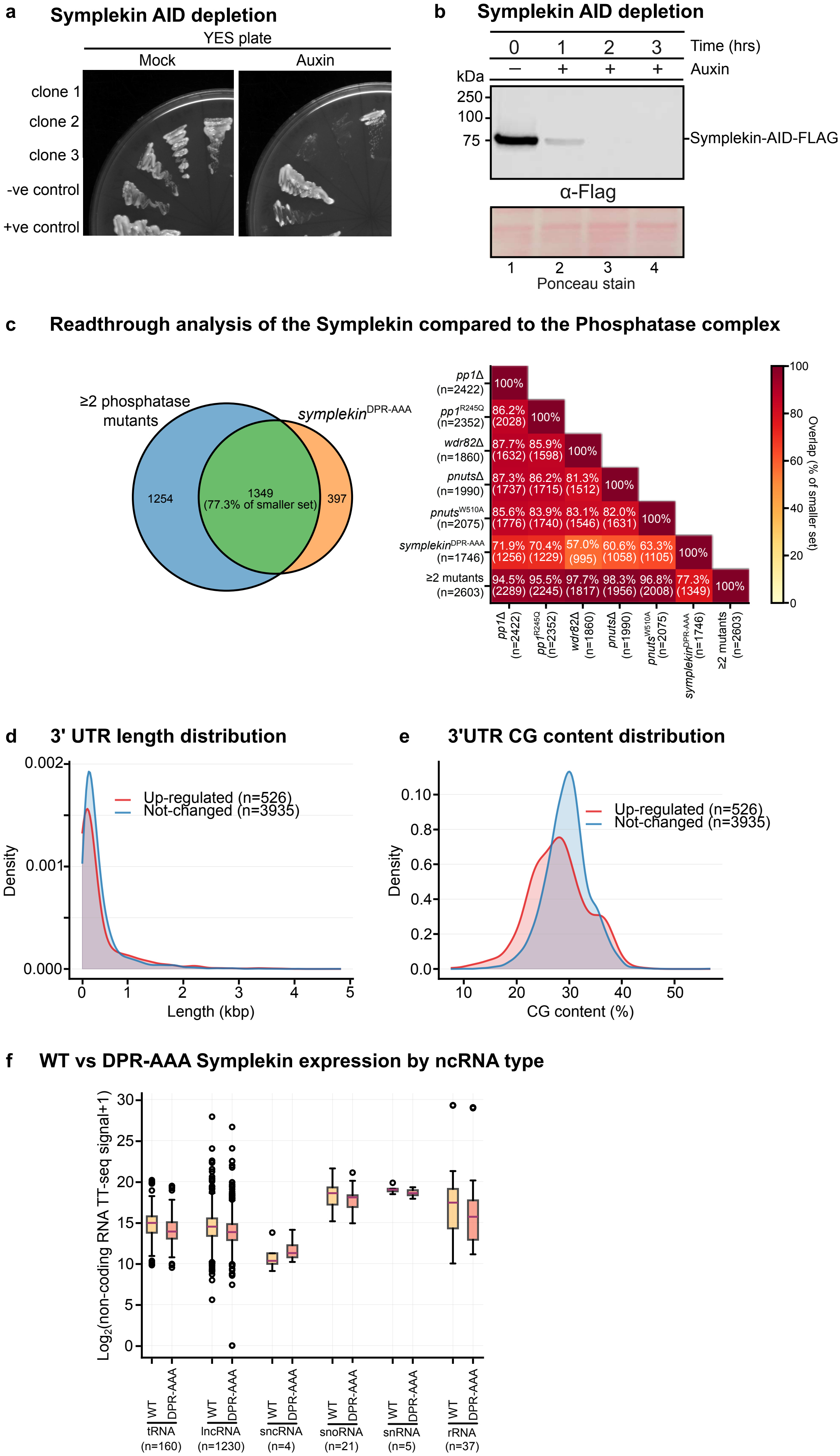

Supplementary Figure 5. Symplekin is essential and its DPR motif is required for correct transcription termination. a Growth assay indicating that depletion of Symplekin-AID results in lethality when grown on auxin plates. **b** Depletion of Symplekin-AID following treatment with 1 mM Auxin. Ponceau stain used as loading control. **c** Overlap between readthrough observed in the PPW mutants and the effect observed in Symplekin$^{DPR\text{-}AAA}$.

d Distribution of 3'UTR lengths which were upregulated at least 1.5-fold in Symplekin$^{DPR\text{-}AAA}$ compared to Symplekin^{WT}, n=526, compared to 3'UTRs that have not been assigned to be changed in control (n=3935).

e Comparison of CG content in 3'UTRs that were upregulated in Symplekin$^{DPR\text{-}AAA}$ compared to Symplekin^{WT}.

f Changes in different classes of non-coding RNAs in Symplekin^{WT} and Symplekin$^{DPR\text{-}AAA}$. Boxes span the interquartile range (25th-75th percentile), the centre line is the median, and whiskers extend to the most extreme value within 1.5 × IQR of the box edges. Circles are outliers beyond the whiskers.

Supplementary Figure 6

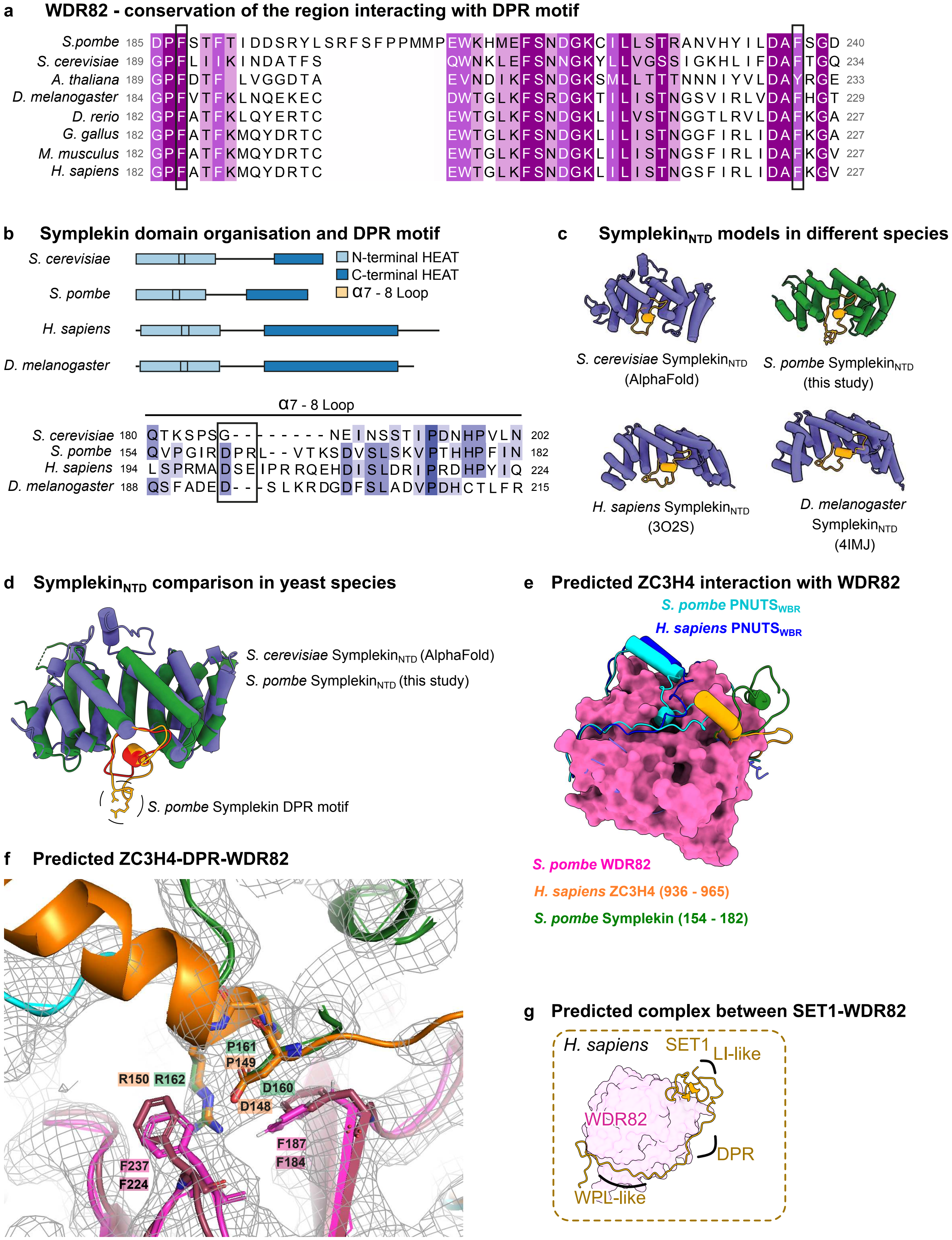

Supplementary Figure 6. Conservation of the WDR82 DPR-binding pocket and of Symplekin$_{NTD}$ across species. **a** Multiple sequence alignment reveals strong conservation within the WDR82 region that forms the pocket engaging DPR motifs, including those found in Symplekin. **b** Comparative domain organisation of Symplekin from multiple organisms, combined with sequence alignment, reveals that the DPR motif is uniquely present in *S. pombe*. **c** AlphaFold predictions of Symplekin$_{NTD}$ structures from various organisms demonstrate that the loop between helices 7 and 8 varies substantially across species and is not evolutionarily conserved. **d** Superimposing the *S. pombe* Symplekin$_{NTD}$ structure with the predicted *S. cerevisiae* NTD reveals substantial divergence, implying that, in budding yeast, the connection between CPA and the phosphatase module is likely mediated by an alternative protein (like Pti1). **e** AlphaFold predictions of the interactions within the ZC3H4-WDR82-PNUTS complex. DPR is provided by ZC3H4, whereas WPL/**I** and L**I**-like by PNUTS. **f** Predicted ZC3H4-DPR-WDR82 binding interface. **g** Structural prediction of WDR82 in complex with SET1 in *H. sapiens*.

Supplementary Figure 7

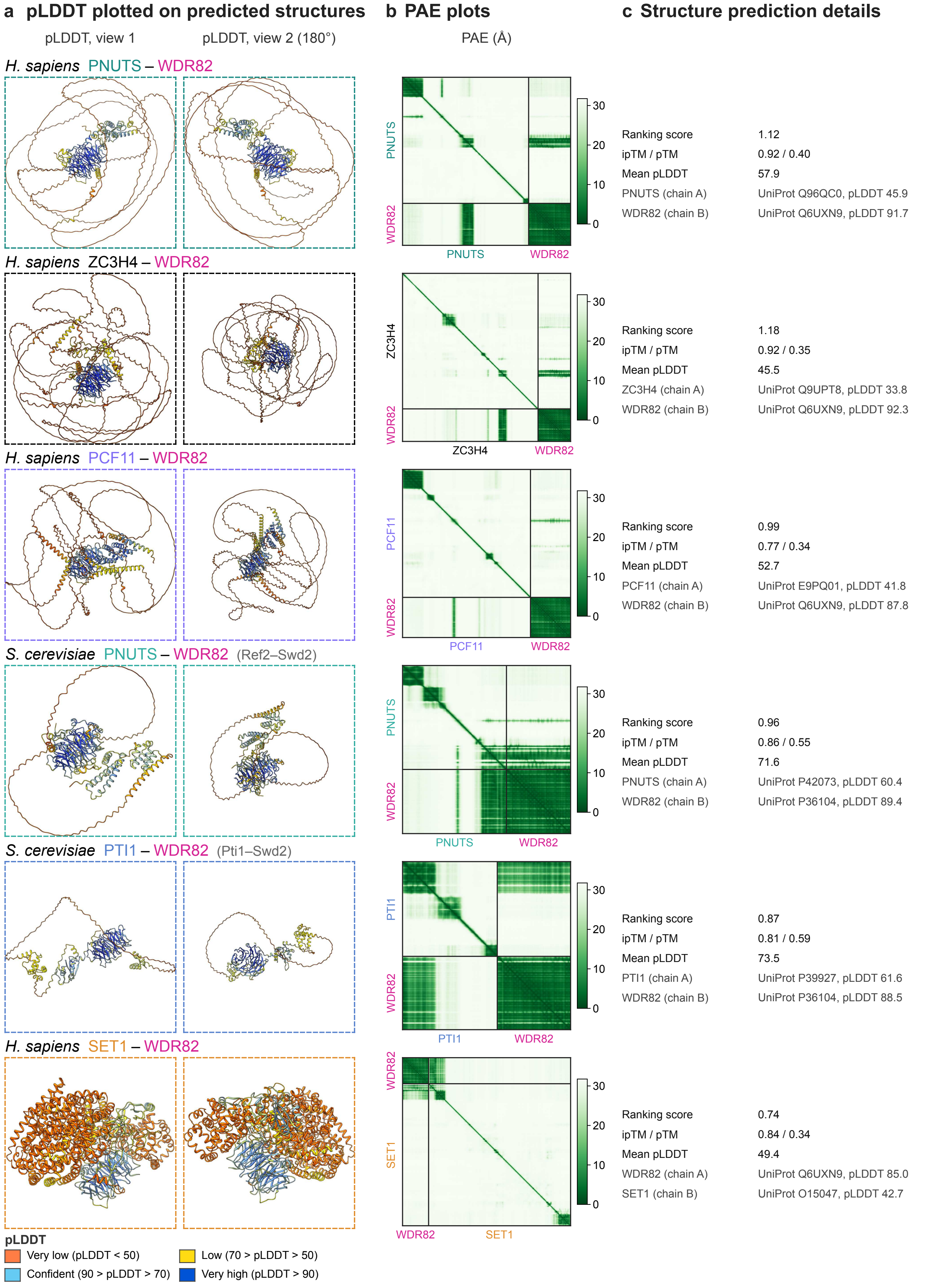

Supplementary Figure 7. Statistics for WDR82 complexes generated with AlphaFold 3. a Predicted structures **coloured by pLDDT, shown in two orientations. b** Predicted aligned error (PAE) plots. **c** Model details, including Ranking score, ipTM, pTM, meanp LDDT, UniProt accessions and per chain statistics.
