# Supplementary material for: WDR82 code coordinates transcription and pre-mRNA 3′-end processing through regulated phosphorylation
Source: Nat Commun. 2026 Sep 15;17:11131. doi: 10.1038/s41467-026-77611-z (PMC13710762; doi:10.1038/s41467-026-77611-z)
Supplement: Supplementary file 2 [file 41467_2026_77611_MOESM2_ESM.pdf]

Description of Additional Supplementary Files

File name: Supplementary Data 1

Description: WDR82 / Swd2 / Swd22 across species — subunits of the three WDR82-family complexes

File name: Supplementary Data 2

Description: Strains and plasmids used in this study

File name: Supplementary Data 3

Description: X-ray data collection, refinement statistics and map density snapshots

File name: Supplementary Data 4

Description: Mass spectrometry results. Data are organised across multiple worksheets
