# Supplementary material for: WDR82 code coordinates transcription and pre-mRNA 3′-end processing through regulated phosphorylation
Source: Nat Commun. 2026 Sep 15;17:11131. doi: 10.1038/s41467-026-77611-z (PMC13710762; doi:10.1038/s41467-026-77611-z)
Supplement: Supplementary file 3 [file 41467_2026_77611_MOESM7_ESM.pdf]

nature portfolio

Peer Review File

WDR82 Code Coordinates Transcription and pre-mRNA 3'-End Processing Through Regulated Phosphorylation

Corresponding Author: Professor Lidia Vasiljeva

This file contains all reviewer reports in order by version, followed by all author rebuttals in order by version.

Version 0:

Reviewer comments:

Reviewer #1

(Remarks to the Author)
This manuscript describes a molecular tour de force on the biochemistry and structural biology of the critical transcriptional elongation/termination factor WDR82. This factor has been associated with different complexes in different eukaryotic models including budding and fission yeast, fruit flies and mammals. This study uses fission yeast, S. pombe as a good interface between simpler and more complex eukaryotes.
This reviewer is non specialist in structural biology so will focus on the key molecular biology results presented.

WDR82 is a well-known component for the critical phosphatase PP1 complex that dephosphorylates RNA Pol II CTD as well as SPT5 Kow domains both of which are associated with a transcription elongation slowdown and gene 3' ends. This is thought to allow time for the 3' end processing activity of CPA to occur and so release polyA+ mRNA and RNA Pol II from the chromatin template and so promote gene transcriptional termination. How WDR82 coordinates this fundamental process is at best only sketchily understood. This new study now adds a lot of critical biochemistry and structural biology to this important process and so substantially advances understanding.

Specific comments mainly to aid reader comprehension.
1) I feel a figure schematic needs to be provided that clearly shows the different gene /protein nomenclature for the different factors involved between SC and SP yeasts, flies and humans that interact with WDR82 and how this leads to different complexes in the different eukaryotes.
2) P5. Be clear that when yeast is referred to S. Pombe is meant.
3) Some comment on why in Figure 1 Pol II CTD Thr4-P is only measured and not CTD Ser2-P which is certainly similarly acted on by PPI at least in S. Cerevisiae.
4) A clearer description of how S. Pombe strains with different PPW-Symplekin mutants were genetically constructed would help non-S. Pombe experts, unaware of the power of S. Pombe genetic analyses.
5) I felt a bit bombarded by some of the acronyms utilised. These sometimes abbreviate proteins that make up a complex (e.g. PPW or APT) or others that refer to a specific aa sequence domain (e.g. Symplekin's DPR motif). Finally, LMWPTP refers to a particular protein fold!

Overall, I feel that this study beautifully demonstrates that the PP1-PNUTS-WDR82 (PPW) complex acts as a key molecular coordinator between transcription elongation/termination and 3'end processing. Especially interesting/important is the data showing that while PNUTS directly activates PPI, WDR82 instead bring PPW to CPA through a direct association with the Symplekin DPR motif, The dominant negative effect of the Symplekin DPR-AAA mutant is especially interesting. The strength of this study is that it combines transcriptomic, biochemical and structural analysis all in one substantial study. Consequently, the data presented is both convincing and important to the field. With minor corrections (mainly clarifications) this paper is surely worthy of publication by Nature Communication.

Reviewer #2

(Remarks to the Author)

This study provides a comprehensive mechanistic and structural characterization of the conserved PP1–PNUTS–WDR82 (PPW) complex and its role in coupling Pol II transcription to pre-mRNA 3′-end maturation. By integrating in vitro reconstitution, X-ray crystallography, and nascent transcriptomics (TT-seq), the authors demonstrate that PNUTS stimulates PP1's phosphatase activity toward Spt5, while WDR82 acts as a critical scaffold bridging the phosphatase to the CPA machinery via the Symplekin DPR motif. The strength of this manuscript lies in its multidisciplinary approach, combining high-resolution structural biology with functional genomics in S. pombe. Notably, the presentation of the first experimental structure of the PNUTS–WDR82–Symplekin–Ssu72 complex is a significant achievement. The proposed model of a 'combinatorial WDR82-binding code' provides a sophisticated framework for understanding how WDR82 integrates diverse transcriptional and RNA processing signals. The study is timely, well-executed, and addresses a fundamental question in gene regulation. While the study is robust and the conclusions are well-supported, there are several issues that should be addressed.

1.Line 78-100: This part can be shortened as it contains too much detail not related to the core of this research.

2.Figure1B: The authors should also examine the status of Thr1-P Spt5 in addition to Thr4-P PolII

3.Line 184-191/Figure 1C: To be fair, these data show only that PPT interacts with PNUTS and WDR82 (PP1/PNUTS and PP1/WDR82 complexes), but do not necessarily show that PP1/PNUTS/WDR82 form a trimeric complex.

4.Line 200-202: The sentence is not complete, missing a verb?

5.Line 270-273/Figure3C: As the recombinant PP1 can be obtained as shown in Figure 1C, the author should add the recombant PP1 to the purified PNUTs/Symplekin/WDR82/Ssu72 complex to clearly show that PPT1 form a complex with these proteins.

6.Figure 3E: It is not clear which lanes in WB correspond to the gel above. Is it for Lane 6,7 and 8? If so, it seems that PP1 binds to PNUTS less when there is WDR82. Please explain.

7.Figure 4B: It is very difficult to understand how PNUTS interacts with WDR82 in the current surface model of WDR82. The authors should revise the figure to clearly show the interactions for YT, WPL/I, LI-like motif. In addition, the authors should visualize the residues of WDR82, which interact with PNUTS.

8.Please consider making a separate section after line 370.

9.Figure 5D: While the MS spectral counts provide quantitative evidence, the authors should perform WB of the IP experiments (V5-Symplekin WT vs. DPR-AAA), probing for PNUTS, PP1, and WDR82.

10.Figure 5E: Please quantify the levels of Thr4-P in the gel.

Reviewer #3

(Remarks to the Author)
The paper by Au et al reports a significant advance in understanding how transcription termination is coupled to 3' end processing by rigourously establishing a new connection between the PP1-PNUTS phosphatase complex and the symplekin subunit of the cleavage polyadenylation apparatus. The authors show in a compelling way that this connection is mediated by binding of PNUTS and symplekin to a common intermediary factor WDR82. They further show that symplekin contacts WDR82 via a DPR motif conserved in other WDR82 binders and that this motif is required in vivo for efficient transcription termination downstream of poly A sites. Throughout the manuscript the results clearly support the authors' conclusions with one exception.
The conclusion that "CPA-mediated PAS selection" (line 247) is regulated by the PPW complex was not adequately justified in my view because it is based solely on evidence of TT-seq nascent RNA coverage not on mapping sites of polyA addition which is usually done using a method such as 3' READS (eg Hoque et al Nat. Methods 10:133, 2013) that ligates a linker to 3' ends of mRNAs within the polyA tail. I did not see in the methods section a clear explanation of how polyA site mapping could be extracted from TT-seq data, but perhaps the authors could elaborate.

Version 1:

Reviewer comments:

Reviewer #1

(Remarks to the Author)

I have looked through this revised ms and the response to reviewers comments (rebuttal) doc.

I feel that all the original comments are thoroughly addressed as are the other two reviewer's comments.

Consequently, I consider this ms to be worthy of Nat Comms publication forthwith.

Reviewer #2

(Remarks to the Author)
The revised manuscript address all of the concerns raised by this reviewer.

Open Access This Peer Review File is licensed under a Creative Commons Attribution 4.0 International License, which permits use, sharing, adaptation, distribution and reproduction in any medium or format, as long as you give appropriate credit to the original author(s) and the source, provide a link to the Creative Commons license, and indicate if changes were made.
In cases where reviewers are anonymous, credit should be given to 'Anonymous Referee' and the source.
The images or other third party material in this Peer Review File are included in the article’s Creative Commons license, unless indicated otherwise in a credit line to the material. If material is not included in the article’s Creative Commons license and your intended use is not permitted by statutory regulation or exceeds the permitted use, you will need to obtain permission directly from the copyright holder.
To view a copy of this license, visit https://creativecommons.org/licenses/by/4.0/

Response to Reviewer Comments

We thank all three reviewers for their careful reading of the manuscript and their constructive comments. We were pleased that the reviewers found our work "a molecular tour de force", " timely, well-executed, and addresses a fundamental question in gene regulation" and overall " substantially advances understanding", which is very encouraging.

The reviewers made excellent suggestions for additional analyses and experiments that we have performed. Below we provide a point-by-point response to each comment. The reviewers' comments are in regular font, and our responses are ***in italics****.*

With the addition of new data and other improvements suggested by the reviewers, we believe we have strengthened the main conclusions of the manuscript and hope to have satisfied the reviewers' concerns.

Reviewer #1

Comment 1: I feel a figure schematic needs to be provided that clearly shows the different gene/protein nomenclature for the different factors involved between SC and SP yeasts, flies and humans that interact with WDR82 and how this leads to different complexes in the different eukaryotes.

We have now compiled a table of the known WDR82-containing complexes (Supplementary Table 2) and updated the text in the manuscript accordingly.

Comment 2: Be clear that when yeast is referred to, S. pombe is meant.

We thank the reviewer for this suggestion. We have revised the manuscript throughout to specify either "fission (S. pombe) or budding yeast (S. cerevisiae)" wherever "yeast" was used ambiguously.

Comment 3: Some comment on why in Figure 1 Pol II CTD Thr4-P is only measured and not CTD Ser2-P which is certainly similarly acted on by PP1 at least in S. cerevisiae.

We have added a sentence clarifying our rationale for using Pol II Thr4-P as the primary readout. Briefly, Thr4-P was used as the primary PP1 substrate readout, as Thr4 dephosphorylation is specifically dependent on PP1 in fission yeast, whereas other CTD residues Ser2-P and Ser7-P/Ser5-P are not affected by PP1 as we reported previously (Kecman et al., 2018 Cell Reports doi: 10.1016/j.celrep.2018.09.007) and instead are

targeted by other phosphatases - Fcp1 and Ssu72 (Heo et al., 2021 Cell Reports doi: 10.1016/j.celrep.2021.109671).

Comment 4: A clearer description of how S. pombe strains with different PPW-Symplekin mutants were genetically constructed would help non-S. pombe experts, unaware of the power of S. pombe genetic analyses.

We have expanded the strain construction section of the Methods to provide a more accessible description of the genetic approaches used, including description of the genomic integration that relies on homologous recombination as well as details on how selection, screening and validation was performed.

Comment 5: I felt a bit bombarded by some of the acronyms utilised. These sometimes abbreviate proteins that make up a complex (e.g. PPW or APT) or others that refer to a specific aa sequence domain (e.g. Symplekin's DPR motif). Finally, LMWPTP refers to a particular protein fold!

We appreciate this feedback. We have added a table of abbreviations to the manuscript to assist readers (Supplementary Table 1) which is now referenced in the manuscript text in the Introduction and Methods sections as well.

Reviewer #2

Comment 1: Line 78-100: This part can be shortened as it contains too much detail not related to the core of this research.

We agree with the reviewer and have now condensed this section of the introduction. The detailed description of CPA subunit composition and the Integrator complex has been reduced, retaining only information directly relevant to the findings presented.

Comment 2: Figure 1B: The authors should also examine the status of Thr1-P Spt5 in addition to Thr4-P Pol II.

We performed this experiment using a Phostag gel to resolve phosphorylated and non-phosphorylated forms of Spt5 and quantify levels of Thr1-P Spt5 relative to non-phosphorylated Spt5 in PPW mutants compared to WT and cells lacking Ssu72 that targets Ser5-P Pol II. As suggested by the reviewer, quantification of Thr4-P has also been performed. The results have been incorporated into the updated Figure 1B and in the text.

Comment 3: Line 184-191/Figure 1C: To be fair, these data show only that PP1 interacts with PNUTS and WDR82 (PP1/PNUTS and PP1/WDR82 complexes), but do not necessarily show that PP1/PNUTS/WDR82 form a trimeric complex.

We agree with the reviewer. We have revised the text accordingly stating that the data we present support interactions between PP1 and PNUTS/WDR82. We do however demonstrate formation of the stable complex between PNUTS and WDR82 and report its structure (Fig S3 and Fig 4).

Comment 4: Line 200-202: The sentence is not complete, missing a verb.

The sentence has been corrected.

Comment 5: Line 270-273/Figure 3C: As the recombinant PP1 can be obtained as shown in Figure 1C, the authors should add the recombinant PP1 to the purified PNUTS/Symplekin/WDR82/Ssu72 complex to clearly show that PP1 forms a complex with these proteins.

We thank the reviewer for this suggestion; however, multiple lines of evidence, including the co-immunoprecipitation data in Figure 1C and the in vitro pulldown in Figure 3E, demonstrate PP1 association with PNUTS and WDR82 and support the formation of a complex among all three subunits. Furthermore, we demonstrate that mutation in conserved PP1 binding motif in PNUTS (pnuts^{W510A} mutant) leads to loss of PNUTS dependent stimulation of PP1 and defective 3'end processing in W510A mutated cells demonstrating functional important of the interaction between PP1 and PNUTS (Fig 1F and Fig 2). Additionally, the mass spectrometry data in Figure 3C further corroborate the presence of all five subunits in the purified complex, with sub-stoichiometric PP1 levels reflecting the known technical limitations of PP1 expression in insect cells rather than an absence of interaction.

Comment 6: Figure 3E: It is not clear which lanes in WB correspond to the gel above. Is it for Lane 6, 7 and 8? If so, it seems that PP1 binds to PNUTS less when there is WDR82. Please explain.

We have now moved the Western blot to align with the corresponding lanes and renumbered the lanes for improved readability. As the pull-downs are qualitative rather than quantitative in nature, we prefer to refrain from quantitative interpretation. Additionally, presence of WDR82 does not affect ability of PNUTS to stimulate PP1 activity (Fig 1E) arguing against WDR82 interfering with PNUTS binding to PP1.

Comment 7: Figure 4B: It is very difficult to understand how PNUTS interacts with WDR82 in the current surface model of WDR82. The authors should revise the figure to clearly show the interactions for YT, WPL/I, LI-like motif. In addition, the authors should visualize the residues of WDR82, which interact with PNUTS.

As suggested by the reviewer we have now generated new version of Figure 4B where specific residues engaged in interactions are clearly labelled.

Comment 8: Please consider making a separate section after line 370.

We agree with the reviewer that this improves clarity. A new section heading has been added here to delineate the mass spectrometry results and focus on Dis2 substrate phosphorylation levels from the subsequent in vivo functional data.

Comment 9: Figure 5D: While the MS spectral counts provide quantitative evidence, the authors should perform WB of the IP experiments (V5-Symplekin WT vs. DPR-AAA), probing for PNUTS, PP1, and WDR82.

We thank the reviewer for this suggestion. Unfortunately, antibodies against S. pombe PNUTS, PP1, and WDR82 are not commercially available, precluding this experiment.

However, the mass spectrometry data in Figure 5D clearly demonstrates loss of the interaction with PPW complex subunits in Symplekin$^{DPR\text{-}AAA}$ compared to in Symplekin^{WT,} while the rest of the Cleavage Polydenyaltion machinery is retained. We therefore consider the MS data to provide sufficient quantitative evidence for this conclusion.

Comment 10: Figure 5E: Please quantify the levels of Thr4-P in the gel.

Quantification is now included above the gels with phosphoisoforms as relative to WT (Fig 5 E).

Reviewer #3

Comment 1: The conclusion that "CPA-mediated PAS selection" (line 247) is regulated by the PPW complex was not adequately justified in my view because it is based solely on evidence of TT-seq nascent RNA coverage not on mapping sites of polyA addition which is usually done using a method such as 3' READS. I did not see in the methods section a clear explanation of how polyA site mapping could be extracted from TT-seq data, but perhaps the authors could elaborate.

We thank the Reviewer for this valid point. The Reviewer is correct that TT-seq does not directly map poly(A) sites: we did not extract PAS positions but instead quantified relative changes in read density across annotated 3'UTRs (the ratio of 3'UTR to gene-body signal), which reflect failed cleavage that could be either due to altered PAS selection or compromised CPSF73/Ysh1 activity. Although our approach reflects altered 3'UTR usage and we agree that definitively mapping PAS selection would require a

direct method and intend to address this in future work using direct RNA nanopore sequencing. We have now modified the text to convey the message more accurately when describing the results and adjusted the conclusions :"We propose that the PPW complex may participate in regulation of the CPA function either contributing to PAS selection or CPSF73/Ysh1 activity " (line 245).
